# A review of *Phyllanthus urinaria* L. in the treatment of liver disease: viral hepatitis, liver fibrosis/cirrhosis and hepatocellular carcinoma

**DOI:** 10.3389/fphar.2024.1443667

**Published:** 2024-08-09

**Authors:** Linhua Liu, Bing Wang, Yibo Ma, Kunhui Sun, Ping Wang, Meifang Li, Junlin Dong, Meirong Qin, Mingshun Li, Chunshan Wei, Ying Tan, Jinsong He, Keying Guo, Xie-an Yu

**Affiliations:** ^1^ NMPA Key Laboratory for Quality Research and Evaluation of Traditional Chinese Medicine, Shenzhen Institute for Drug Control, Shenzhen, China; ^2^ State Key Laboratory of Chemical Oncogenomics, Institute of Biopharmaceutical and Health Engineering, Shenzhen lnternational Graduate School, Tsinghua University, Shenzhen, China; ^3^ Department of Liver Disease, Shenzhen Traditional Chinese Medicine Hospital, The Fourth Clinical Medical College of Guangzhou University of Chinese Medicine, Shenzhen, China; ^4^ Department of Biotechnology and Food Engineering, Guangdong-Technion Israel Institute of Technology, Shantou, China

**Keywords:** Phyllanthus urinaria L., liver disease, viral hepatitis, liver fibrosis/cirrhosis, hepatocellular carcinoma

## Abstract

Due to the pathological production of liver disease in utility particularly complexity, the morbidity and mortality of liver disease including viral hepatitis, liver fibrosis/cirrhosis and hepatocellular carcinoma (HCC) are rapidly increasing worldwide. Considering its insidious onset, rapid progression and drug resistance, finding an effective therapy is particularly worthwhile. *Phyllanthus urinaria* L. (*P. urinaria*), an ethnic medicine, can be applied at the stages of viral hepatitis, liver fibrosis/cirrhosis and HCC, which demonstrates great potential in the treatment of liver disease. Currently, there are numerous reports on the application of *P. urinaria* in treating liver diseases, but a detailed analysis of its metabolites and a complete summary of its pharmacological mechanism are still scarce. In this review, the phytochemical metabolites and ethnopharmacological applications of *P. urinaria* are summarized. Briefly, *P. urinaria* mainly contains flavonoids, lignans, tannins, phenolic acids, terpenoids and other metabolites. The mechanisms of *P. urinaria* are mainly reflected in reducing surface antigen secretion and interfering with DNA polymerase synthesis for anti-viral hepatitis activity, reducing hepatic stellate cells activity, inflammation and oxidative stress for anti-liver fibrosis/cirrhosis activity, as well as preventing tumor proliferation, invasion and angiogenesis for anti-HCC activity via relevant signaling pathways. Accordingly, this review provides insights into the future application of natural products in the trilogy of liver diseases and will provide a scientific basis for further research and rational utilization of *P. urinaria*.

## 1 Introduction

Liver disease, a globally widespread disease accounting for 4% of the global death toll each year, is a serious challenge to people’s health in different regions with numerous risk factors such as viral infections, excessive alcohol consumption, unhealthy diet, and drug injury (Devarbhavi et al., 2023). The early symptoms of liver disease may not be obvious, whereas the symptoms of abdominal pain, fatigue, jaundice and other concomitant symptoms are present following the aggravation of the infection (Neshat et al., 2021). The progression of liver disease usually passes through three stages including viral hepatitis, liver fibrosis/cirrhosis and hepatocellular carcinoma (HCC), which is called the trilogy of liver disease (Zeng et al., 2023). Infection with Hepatitis B virus (HBV) and Hepatitis C virus (HCV) is a known risk factor for HCC and the major contributor to the progression of chronic viral hepatitis to liver fibrosis/cirrhosis, ultimately leading to HCC (Fan et al., 2020). While vaccination and anti-viral therapy offer potential in decreasing the incidence of viral hepatitis, the emergence of resistance from prolonged use presents a significant obstacle to eradicating the disease (Thomas, 2019). Furthermore, the lack of effective supervision in vaccination, negligence in ensuring injection safety during the vaccination process, potential side effects of oral medications like headaches and fever, and the economic burden of long-term drug use all highlight the ongoing difficulty in finding a definitive cure for the progression of liver disease (Sarin et al., 2020). Consequently, there is a pressing demand for the exploration and development of alternative therapies to comprehensively address the trilogy of liver disease.

Recently, there has been a growing focus on natural products as complementary and alternative medicine for both preventing and treating liver diseases (Calixto et al., 1998). Natural products have potential biotherapy activities including anti-viral, balancing oxidative stress and anticancer effects, protecting the liver, and are widely used in the treatment of liver diseases, thus they are more readily available and widely used in the treatment of diseases (Xu et al., 2024). The *Phyllanthus* encompasses a vast array of species, with over 1,000 species contain more than 500 bioactive metabolites. They have been reported to included alkaloids, flavonoids, lignans, phenol, tannins, and terpenes, as well as others. Among them, about 16 most commonly used *Phyllanthus* species have a significant impact on liver disease*, contains Phyllanthus emblica L., Phyllanthus niruri L., Phyllanthus amarus Schumach,* and *Phyllanthus urinaria L.,* as well as others. *Phyllanthus emblica* L. extract metabolites have been shown to reduce oxidative stress injuries and suppress inflammatory responses, resulting in beneficial therapeutic effects on various liver diseases such as liver injury, fatty liver, viral hepatitis, and liver fibrosis (Jantan et al., 2019). Additionally, the 50% methanol extract of *P. niruri* L. has demonstrated hepatoprotective effects against the progression of nonalcoholic fatty liver disease (NAFLD) in rats. This extract also led to reduced visceral adiposity, improved liver enzyme abnormalities, and decreased hepatic lipid peroxidation and fat accumulation (Kaur et al., 2017). Various species of *Phyllanthus*, including *P. amarus* L. (Reddy et al., 2018), *P. niruri* L. (Venkateswaran et al., 1987), *Phyllanthus nanus* L. (Lam et al., 2006), and *Phyllanthus acidus* L. (Lv et al., 2014) have demonstrated inhibitory effects on hepatitis viruses through *in vitro*, *in vivo* experiments (Thyagarajan et al., 1988), and in clinical trials (Mandal and Hazra, 2023). Meanwhile, *Phyllanthus urinaria* L. (*P. urinaria*) is a well-known member of the *Phyllanthus* family recognized for its therapeutic potential in treating liver disease. Numerous studies have emphasized its effectiveness in managing HBV infection and its utilization in conjunction with traditional medicines for liver disease treatment in ethnomedicine (Qi et al., 2014). Moreover, *P. urinaria* has exhibited hepatoprotective properties by reducing elevated levels of alanine aminotransferase (ALT) and aspartate aminotransferase (AST) in animal models of liver injury (Geethangili and Ding, 2018).

**FIGURE 1:**
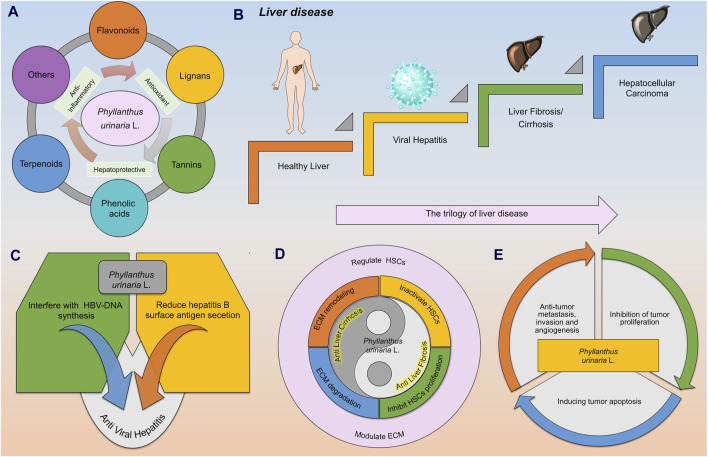
The metabolites of *Phyllanthus urinaria* L. (*P. urinaria*) have been identified for their anti-hepatitis properties, potential in reversing liver fibrosis/cirrhosis, and treatment of HCC. **(A)** The metabolites of *P. urinaria* L. include flavonoids, lignans, tannins, phenolic acids, terpenoids, and others (coumarins, steroids, and alkaloids). **(B)** In the progression of liver disease, a healthy liver can be impacted by a hepatitis virus, leading to conditions like liver fibrosis/cirrhosis and HCC. **(C)** *P. urinaria* combats viral hepatitis by interfering with HBV DNA synthesis and reducing the secretion of hepatitis B surface antigen (HBsAg). **(D)** *P. urinaria* shows properties that can help combat liver fibrosis/cirrhosis by regulating hepatic stellate cells (HSCs) and improving extracellular matrix (ECM) function. **(E)** *P. urinaria* exhibits effects against HCC by inhibiting tumor proliferation, promoting tumor apoptosis, and suppressing tumor invasion, metastasis, and angiogenesis.

The literature lacks a comprehensive on the treatment of the trilogy of liver diseases by *P. urinaria*. This review aims to provide a comprehensive summarized of the metabolites of *P. urinaria* and their therapeutic effects on liver diseases, specifically focusing on anti-viral hepatitis, anti-liver fibrosis/cirrhosis, and inhibition of HCC. This review includes findings from *in vitro*, *in vivo* experiments as well as insights from clinical applications, as illustrated in Figure 1. Additionally, it provides the morphology, geographic distribution, extraction method, and future challenges in the clinical utilization of *P. urinaria*. The metabolites can be found in Supplementary Table S1 and Supplementary Figure S1–S6, a thorough understanding of the therapeutic mechanisms of *P. urinaria* is essential for optimizing its bioactivity and bioavailability.

## 2 Methods

Literature for this review was gathered from various search engines and databases, including Web of Science, PubMed, SciFinder, Science Direct, Embase, Springer Link, and EBSCO. Articles published before May 2024 on the phytochemical and ethnopharmacological applications of *P. urinaria*, its use in treating viral hepatitis, liver fibrosis/cirrhosis, and HCC were reviewed. The search terms used were “*Phyllanthus urinaria* L.”, “*Phyllanthus*”, “*P. urinaria*”, “Liver disease”, “Viral hepatitis”, “Liver fibrosis/cirrhosis”, ‘Hepatocellular Carcinoma’. Full-text access had no time limit, and there were no language restrictions on the literature. Additional articles were gathered through literature citations, related sources, and journal websites to ensure comprehensive coverage.

## 3 Results

### 3.1 Botanical description, distribution and quality control

#### 3.1.1 Botanical characteristics of *Phyllanthus urinaria* L.

**FIGURE 2:**
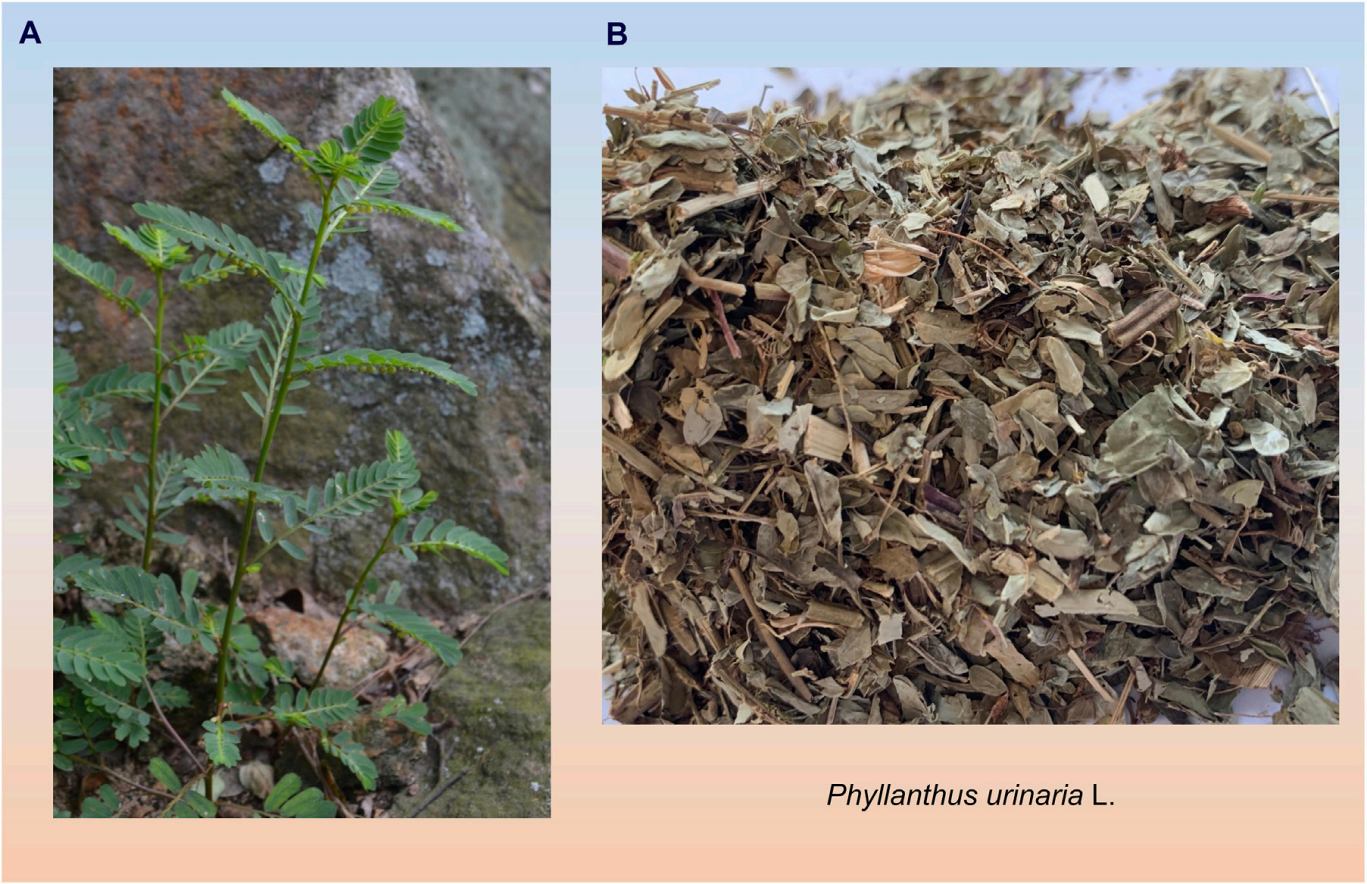
Morphology and medicinal parts of *P. urinaria* are depicted in photographs. **(A)** Photographs of the whole botanical drug of *P. urinaria* **(B)** Photographs of the medicinal use of *P. urinaria*.

The botanical classification of *P. urinaria* includes its generic name and unique characteristics, an annual plant categorized under the *Plantae*, *Angiospermae*, *Magnoliopsida*, *Malpighiales*, *Phyllanthaceae*, and *Phyllanthus*. The plant features dark reddish-brown fibrous roots, upright stems ranging from 10–60 cm with a brown-red surface, leaves arranged in two rows, oval-shaped capsules that are oblate-spherical and sessile, female flowers are located at the proximal end, while male flowers are found at the distal end (Rossignol et al., 1987). The entire plant including leaves, stems, fruits, and roots, is utilized for medicinal purposes, as depicted in Figure 2, its distinct characteristics include brown roots, leaves, and fruits, with small easily broken brown leaves, and unique microscopic features in roots, leaves, and powder (Gama et al., 2016). This plant is valued by diverse ethnic groups, with its medicinal properties acknowledged in various traditional healing practices in countries like China, India, Indonesia, Japan, and the United States. For example, in India, it is commonly known as “shatter stone”, “leaf flower”, and “chamber bitter”, plays a significant role in ayurvedic treatments for a range of ailments (Sharma et al., 2012). In Brazil, it is referred to as ‘arrebenta pedra’ and is utilized in local folk medicine to address liver and kidney diseases (Calixto et al., 1998). In China, *P. urinaria* is known as “yexiazhu”, “pearl grass”, “leaf pearl”, or “dragon pearl grass” due to its red and green fruits, and it is used medicinally for conditions such as diarrhea, enteritis, jaundice, and liver ailments, with Traditional Chinese Medicine (TCM) recognizing its liver-protective properties (Du et al., 2018).

#### 3.1.2 Distribution and identification of *Phyllanthus urinaria* L.

*P. urinaria*, commonly found in tropical and subtropical regions such as Asia, Africa, and South America, has been traditionally used for treating a variety of conditions including diabetes (Gunawan-Puteri et al., 2012), heart disease (Chularojmontri et al., 2005; Chularojmontri et al., 2009), bone and joint issues (Buddhachat et al., 2017), inflammation, and neuropathic pain (Dias et al., 1995). *P. urinaria* is vulnerable to contamination by common adulterants, Fang et al. (Fang et al., 2022) employed molecular marker technology to enhance the genome resources of *P. urinaria*, identifying genetic differences through regions such as petA-psbJ, trnS-GCU-trnG-UCC, and trnT-UGU-trnL-UAA. They utilized the intergenic spacer (IGS) to distinguish between different species within the genus and species of *P. urinaria*. Viaene et al. (Viaene et al., 2015) used reversed-phase high performance liquid chromatography with ultraviolet detection (RP-HPLC-UV) to create fingerprints for distinguishing *P. urinaria* from *Phyllanthus,* they recommended the use of soft independent modeling of class analogy (SIMCA) to effectively gather sensitive and specific information on various herbal medicines. Orman and colleagues (Orman et al., 2023) developed a method called Quantitative assessment of multi-components by single markers (QAMS) to monitor the quality of *P. urinaria*. This approach utilizes rutin as a reference standard. The relative correction factors (RCFs) for isoquercitrin, geraniin, and phyllanthin were calculated to assess the levels of metabolites in *P. urinaria* sourced from various locations and harvested at different time points.

### 3.2 The trilogy of liver disease

**FIGURE 3:**
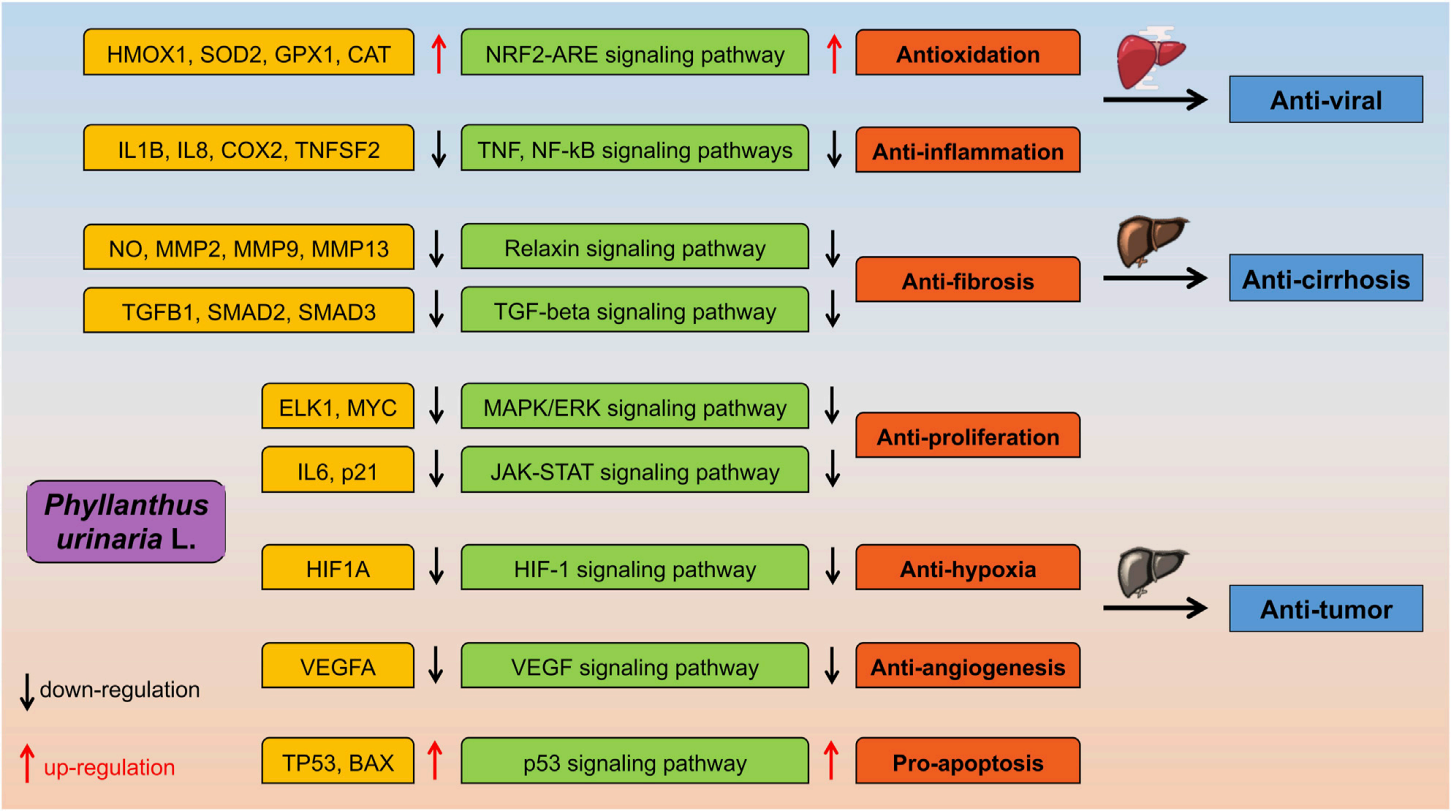
The proposed molecular mechanisms of *P. urinaria* suggest its potential anti-viral, anti-fibrosis/cirrhosis, and anti-tumor activities.

Viral hepatitis, resulting from infection with hepatitis viruses, is a significant contributor to liver fibrosis/cirrhosis, and HCC. The types of viral hepatitis, namely, A, B, C, D, and E, are determined by the specific virus causing the infection (Zeng et al., 2021). Worldwide, the primary risk factors for HCC are HBV and HCV infections. HBV and HCV are classified as human carcinogens, with HBV being the predominant cause in Asia and Africa, while HCV is more prevalent in HCC cases in Europe and the Americas (Villanueva, 2019). HBV, a partially double-stranded DNA virus, contains the oncoprotein hepatitis B virus x protein (HBx) in its genome, which plays a crucial role in driving the progression of HCC, sometimes bypassing the end-stage liver disease phase (Ringelhan et al., 2017). Liver fibrosis is a common pathological process in the progression of various chronic liver diseases to cirrhosis. Early diagnosis and treatment of liver fibrosis can prevent the advancement of chronic liver diseases to cirrhosis and HCC, leading to improved outcomes. Viral hepatitis is a well-known risk factor for liver fibrosis, as chronic liver inflammation can result in widespread fibrosis and eventual cirrhosis. Liver fibrosis occurs when the liver is damaged, leading to the deposition of extracellular matrix proteins during the body’s repair process (Caligiuri et al., 2021). The development of liver fibrosis/cirrhosis, and subsequent HCC (Gnyawali et al., 2022) from hepatitis B is associated with DNA damage caused by oxidative stress resulting from persistent inflammation, hepatocyte senescence, and alterations in the tumor microenvironment (Karakousis et al., 2020). The HBx gene transactivates the virus during infection and plays a role in influencing the proliferation, differentiation, and apoptosis of hepatocytes through protein interactions, various signaling pathways, and induction of immune imbalance. This is a crucial factor in the development of HCC. The HBx protein is involved in regulating cccDNA, controlling HBV-DNA damage, and disrupting HBV replication within cells. The stability of HBx is strengthened by TNF-α, NF-kB, Wnt/β-catenin, and other pro-inflammatory pathways, which in turn regulate the proliferation and apoptosis of HCC cells (Han et al., 2014). Inflammation plays a crucial role in the progression of liver disease through various mechanisms, the inflammatory environment can contribute to the development of liver fibrosis by activating TNF-β. Persistent inflammation can lead to alterations in ROS levels, which in turn impact oxidation, extracellular matrix (ECM), and collagen synthesis, increasing the likelihood of liver cirrhosis. Furthermore, inflammation and excessive oxidative stress can disrupt the liver microenvironment, triggering abnormal molecular signaling pathways like JNK, PPAR, and AMPK that promote tumor proliferation, metastasis, and progression to HCC. Overall, the interconnected signaling pathways and responses triggered by inflammation, oxidative stress, and microenvironmental changes are key features in the progression of liver diseases, as shown in Figure 3, with both innate and adaptive immunity playing a significant role in the development of chronic liver diseases. (Zhang et al., 2023).

### 3.3 Application status of *Phyllanthus urinaria* L. in anti-viral hepatitis

#### 3.3.1 Epidemiology and treatment of viral hepatitis

Research indicates that by 2020, deaths from liver diseases linked to viral hepatitis B and C are expected to exceed 1.1 million, surpassing those caused by malaria and Acquired Immune Deficiency Syndrome (Devarbhavi et al., 2023). The majority of these deaths are attributed to hepatitis B, with China, India, and Nigeria carrying the highest burden of the disease (Sarin et al., 2020). Chronic hepatitis B (CHB) can manifest in various ways, including asymptomatic cases, liver fibrosis, advanced cirrhosis, and HCC. It is estimated that there are 296 million individuals living with CHB (Dusheiko et al., 2023), the prevalence of hepatitis C is similar to that of hepatitis B, with a global rate of 0.7% in 2020. Countries with the highest burden of hepatitis C include China, Pakistan, and India (Polaris Observatory, 2022). While preventive vaccination has limited impact on reducing hepatitis B burden (Yuan et al., 2022), nucleotide analogs and antiviral drugs have shown efficacy in treatment, although they may not completely eliminate the risk of HCC development (Roade et al., 2021). Hence, there is a necessity to explore additional complementary and alternative antiviral therapies. Baruch Blumberg conducted both *in vitro* and *in vivo* experiments using an extract from *P. niruri* L., demonstrating a preliminary inhibitory effect on HBV-DNAP (Venkateswaran et al., 1987). Additionally, Thyagarajan conducted clinical trials using *P. amarus* L. (Thyagarajan et al., 1988). Experimental data suggests that *Phyllanthus* has the potential to inhibit HBV virus replication, reverse transcriptase, and DNA polymerase. Initial clinical trials have demonstrated positive outcomes of *P. urinaria* against HBV (Chen and Zhang, 2023). The pharmacological activities of *P. urinaria*, including extraction methods, mechanisms of action, and effects, are summarized in Supplementary Table S2.

#### 3.3.2 The metabolites of anti-viral hepatitis

The anti-viral hepatitis metabolites of *P. urinaria* are detailed in Supplementary Table S1, with its biological activities primarily linked to antioxidant properties stemming from hydroxyl groups (Sarin et al., 2014). The use of the entire plant for medicinal purposes or extracting it with polar solvents like methanol, ethanol, or aqueous has demonstrated positive physiological effects. Various extracts of *P. urinaria*, including aqueous, methanol, ethanol, and acetone, have exhibited anti-bacterial, anti-diabetic, anti-malarial, antioxidant, and anti-tumor properties, suggesting potential benefits for treating conditions such as viral infections, jaundice, hypertension, diabetes, and cancer. For example, research by Yang et al. (Yang et al., 2005) revealed that methanol, ethanol, and acetone extracts of *P. urinaria* could hinder HSV-2 infection at the early stages. HSV-2, a large double-stranded DNA virus causing herpes infections, was effectively inhibited by the aqueous extract of *P. urinaria*, with metabolites like trigalloylglucopyronoside playing a key role in preventing virus entry and disrupting host protein manipulation (Tan et al., 2013). Ellagitannins (1-Galloyl-3,6-hexahydroxydiphenoyl-4-O-brevifolin-carboxyl-β-D-glucopyranose, geraniin, corilagin) isolated from the ethanol extract of *P. urinaria* were found to inhibit Epstein-Barr virus (EBV) activity effectively (Zeng et al., 2021). Furthermore, the methanol and chloroform extracts of *P. urinaria* demonstrated significant inhibition of *Helicobacter pylori* infection (Lai et al., 2008), potentially through the regulation of the NF-κB pathway by *P. urinaria* to reduce inflammation. *P. urinaria* is utilized for treating hepatitis. Various metabolites such as trimethyl-3,4-dehydrochebulate, methyl gallate (Fang et al., 2008), gentisic acid 4-O-β-D-glucopyranoside, syringin, ascorbic acid, arbutin, brevifolin carboxylic acid, gallic acid, and protocatechuic acid (Xu et al., 2007) have demonstrated significant biological activity in inhibiting tyrosinase (Hu et al., 2014). Moreover, metabolites like cucurbic acid, α-methyl cucurbate (Urakawa et al., 2004), and phyllanthurinolactone, a glycoside metabolite isolated from *P. urinaria*, have shown promise for the development of bioactive molecular probes. Guo et al. (Guo et al., 2021) utilized mechanochemical-assisted extraction (MCAE) to successfully isolate and identify ellagic acid in the ethanol extract of *P. urinaria*, achieving a rate of 10.2 mg/g. Additionally, gallic acid ethyl ester (GAEE) (Santos et al., 1999) was detected in the ethanol extract of *P. urinaria*, exhibiting analgesic and other effects (Paulino et al., 1999).

#### 3.3.3 Anti hepatitis C

Hepatitis C is caused by a single-stranded RNA virus, Chung et al. (Chung et al., 2016a) demonstrated that the acetone extract of *P. urinaria*, a monoterpene lactone named loliolide, can inhibit HCV infection in a dose-dependent manner. Subsequent research by Chung et al. (Chung et al., 2016b) revealed that (4R, 6S)-2-dihydromenisdaurilide (DHMD), isolated from the acetone extract of *P. urinaria*, has the potential to treat hepatitis C by inhibiting HCV infection in Huh7 cells and preventing HCV entry into cells during the initial infection stage. Interestingly, DHMD exhibits selective inhibition towards HCV by targeting viral glycoprotein E1/E2, hindering glycoprotein-receptor interaction and conformational changes. This suggests DHMD’s potential as a small molecule inhibitor, particularly for application in transplantation settings. Another metabolite, corilagin, identified from the methanol extract of *P. urinaria*’s whole plant, has also shown effectiveness in inhibiting HCV activity. The mechanism of action may involve corilagin binding to HCV N53 protease, forming an enzyme-inhibitor complex that can alter or deactivate the enzyme’s activity structure (Yue et al., 2005).

#### 3.3.4 Interfere with HBV DNA synthesis

In patients with Hepatitis Be antigen (HBeAg) positivity, higher HBV DNA levels in individuals with CHB are linked to a high risk of liver fibrosis and HCC (Liu et al., 2014). The analytical review conducted by Chen et al. (Chen and Zhu, 2013) revealed that *P. urinaria* inhibitory effect on plasma DHBV DNA could reach to over 45%. Moreover, ellagic acid, a phenolic metabolite, demonstrated a protective effect on HBV infection by interfering the secretion of HBeAg rather than interfering with the secretion of HBV DNA, highlighting the significance of quantitatively evaluating HBV DNA in future research. (Aishwarya et al., 2021). Shin et al. (Shin et al., 2005) isolated ellagic acid from *P. urinaria* extracts and demonstrated reduced HBeAg secretion in HepG2 2.2.15 cells post-treatment. Kang et al. (Kang et al., 2006) conducted *in vivo* experiments on HBeAg-producing transgenic mice, indicating that ellagic acid can boost immune responses, decrease HBeAg levels, and enhance cytokine release, thereby confirming its effectiveness against hepatitis B.

#### 3.3.5 Reduce hepatitis B surface antigen secretion

*P. urinaria* demonstrates a strong anti-HBV effect, with phenolic acid, and other metabolites playing a crucial role in its anti-viral properties. Extracts and metabolites from *P. urinaria* have been found to reduce the secretion of HBsAg and HBeAg, as well as decrease serum enzyme levels (Upadhyay and Tiwari, 2023). Monitoring HBeAg and HBsAg levels is essential during anti-viral treatment to evaluate treatment efficacy and disease prognosis due to the burden of HBsAg leading to T cell and B cell depletion (Loggi et al., 2022). Wu et al. (Wu et al., 2015) utilized a recombinant eukaryotic expression plasmid pHBV1.1 to transfect HepG2 cells and establish an *in vitro* model of HBV for assessing the direct anti-viral impact of *P. urinaria* on HBV. Their study revealed that *P. urinaria* extract at concentrations of 0.8 and 0.2 g/L significantly interfered with HBsAg levels. Lamivudine (LMV), a common nucleotide analog for hepatitis B treatment, often leads to drug resistance in 80% of patients after 5 years of treatment. Lam et al. (Lam et al., 2006) engineered a mutant of HBV P protein and introduced it into HepG2 cells to create an *in vitro* model of LMV resistance. Exposure to varying concentrations of *P. urinaria* aqueous extract resulted in a dose-dependent decrease in HBsAg and HBcAg secretion levels. This effect may be attributed to the activation of immunity and induction of COX-2 by *P. urinaria* extract through the ERK1/2 and JNK signaling pathways, leading to increased IL-6 mRNA expression and subsequent interfere of HBV replication. Liang et al. (Liang et al., 2019) identified 16 phenolic compounds in the ethanol extract of *P. urinaria* and evaluated their ability to inhibit HepG2.2.15 cells *in vitro*. The results showed that emodin-8-O-β-D-glucopyranoside, catechin, 3-O-methylgallic acid, ethyl gallate, and protocatechuic acid significantly reduced HBsAg secretion, potentially due to their antioxidant properties. Conversely, Fu et al. (Fu et al., 2023) utilized network pharmacology, *in vitro* experiments, protein interaction networks, and functional enrichment analysis to identify key metabolites in *P. urinaria*, such as gallic acid, kaempferol, quercetin, corilagin, and rutin. The signaling pathways are mainly related to TNF, VEGF, and JAK/STAT. *In vitro* assays have shown that *P. urinaria* extract can reduce HBsAg, HBeAg levels in a dose-dependent and time-dependent manner. This effect may be linked to the increased expression of NFE2L2 and HMOX1 proteins by *P. urinaria*.

#### 3.3.6 Clinical applications

A systematic review indicated that *P. urinaria* had beneficial effects on CHB and improved liver function. The findings revealed a decrease in serum HBsAg secretion in patients treated with *P. urinaria* compared to those receiving a placebo. Moreover, *P. urinaria* was found to be more effective in interfering HBV DNA synthesis and reducing HBeAg levels compared to other botanical drug treatments, as well as superior to interferon monotherapy (Liu et al., 2001a). In a separate systematic review focusing on the treatment of CHB using Chinese herbal medicine, it was found that *P. urinaria* showed a comparable therapeutic effect to interferon. Additionally, *P. urinaria* demonstrated a significant impact in reducing HBeAg levels and interfering with HBV DNA synthesis (Liu et al., 2001b). In a meta-analysis of 53 randomized controlled trials, *P. urinaria* demonstrated superior therapeutic effects compared to interferon alone. *Phyllanthus urinaria* showed OR values of 2.05, 2.17, and 3.7 in interfering with HBV DNA synthesis, lowering HBeAg levels, and improving abnormal ALT values, indicating its potential anti-HBV activity (Zhang et al., 2010). In a clinical trial conducted by Xing et al. (Xing et al., 2020), the Bushen Qingtou prescription, a TCM compound, was administered to CHB patients with normal ALT levels. The study involved 395 patients in a 96-week trial, where those receiving oral TCM experienced a significant reduction in HBV DNA levels by 4 log10 IU/mL at week 48% and 10.05% at week 96, as opposed to 2.55% in the placebo group. These findings suggest that the TCM compound is more effective in decreasing HBV DNA and HBeAg values, potentially through *P. urinaria*’s modulation of host immune function. In a phase II clinical trial by Chan et al. (Chan et al., 2003), varying doses of *P. urinaria* extract were administered to patients with HBeAg-positive status, elevated ALT levels, and high HBV DNA levels for 24 weeks. The results indicated a reduction in HBV DNA levels in the treatment group compared to the placebo group.

### 3.4 Application status of *Phyllanthus urinaria* L. in anti-liver fibrosis/cirrhosis

#### 3.4.1 Epidemiology and treatment of liver fibrosis/cirrhosis

The age-standardized rate of cirrhosis decreased from 25.7 per 100,000 in 1990 to 25.3 per 100,000 in 2019, with a higher prevalence among men than women. Cirrhosis is characterized by liver damage and fibrosis, the primary causes of liver fibrosis/cirrhosis include HBV and HCV infections, nonalcoholic steatohepatitis, and alcoholism, with an increasing incidence of HCV and alcoholism. Chronic liver damage often results from a combination of factors such as chronic viral hepatitis, alcohol consumption, obesity, and others, leading to the development of cirrhosis (Wang et al., 2024). Patients with compensated cirrhosis have a 4.7 times higher risk of death compared to the general population, which increases to 9.7 times higher in those with decompensated cirrhosis. The economic impact of cirrhosis and its complications, such as hemorrhage and ascites, is significant worldwide. Therefore, research on novel drug therapies for liver fibrosis/cirrhosis remains a crucial area of study (Ge and Runyon, 2016). The progression of liver fibrosis/cirrhosis involves various processes including the activation of Hepatic Stellate Cells (HSCs), apoptosis of liver parenchymal cells, capillarization of liver sinusoidal endothelium, epithelial-mesenchymal transition (EMT), and ECM deposition (El Awdan and F. Asaad, 2021). HSCs activation, marked by increased expression of cell phenotype markers like α-SMA and collagen, plays a central role in this progression (Zhang et al., 2022). Treatment with antiviral agents and anti-inflammatory medications can be beneficial in the prevention and management of liver fibrosis and cirrhosis.

Various approaches include inhibiting HSCs activation, collagen fiber deposition, promoting ECM matrix protein degradation, and antagonizing cytokines involved in liver fibrosis. While anti-viral therapy can slow down the progression of liver fibrosis/cirrhosis, its effectiveness is limited in preventing cirrhosis from advancing to HCC (Chang et al., 2010). Currently, there are no approved drugs specifically designed for liver fibrosis treatment, emphasizing the necessity for diverse strategies to address the intricate pathology of liver cirrhosis. Zhao et al. (Zhao et al., 2022) utilized systems biology and network informatics to pinpoint targets and key metabolites in natural drugs for liver fibrosis treatment. KEGG analysis unveiled connections with inflammation and energy metabolism, with drug targets associated with EGFR, PTGS2, ICAM1, TNF, IL-2. Excessive oxidative stress can activate HSCs, and Li et al. (Li et al., 2023) suggest that TCM can regulate oxidative stress to alleviate liver fibrosis. The pharmacological activities of *P. urinaria* are summarized in Supplementary Table S3, detailing the mechanisms of action and effects.

#### 3.4.2 The metabolites of anti-liver fibrosis/cirrhosis

The anti-liver fibrosis/cirrhosis metabolites of *P. urinaria* is detailed in Supplementary Table S1. Flavonoids, known for their antioxidant properties, have been successfully extracted from *P. urinaria*. With the help of ESI-MS and NMR techniques to isolate various flavonoids, such as astragalin and quercetin 3-rutinoside, from the methanol extract of *P. urinaria*. They also discovered a new flavonoid named urinariaflavone (Thanh et al., 2014). *P. urinaria*, commonly employed in treating inflammatory conditions, is known for its heat-clearing, diuretic, liver-calming, and eyesight-improving effects. Metabolites like rhamnocitrin, quercitrin, and rutin (Fang et al., 2008) have demonstrated anti-inflammatory properties. Deng et al. (Deng et al., 2012) used ultraviolet capillary zone electrophoresis (CZE) and electrochemical detection (ED) to accurately identify active ingredients like quercitrin and rutin in *P. urinaria* extract. Analysis through NMR, HR-ESI-MS, and IR techniques revealed the presence of four flavonoids in the ethanol extract of *P. urinaria*, including quercetin 3-O-α-L-rhamnopyranoside, quercetin, quercetin 3-O-α-L-(2,4-di-O-acetyl)rhamnopyranoside-7-O-α-L-rhamnopyranoside, and quercetin 3-O-α-L-(3,4-di-O-acetyl)rhamnopyranoside-7-O-α-L-rhamnopyranoside (Wu et al., 2013). Among these metabolites, kaempferol 7-methyl ether, quercetin 3-O-β-D-glucoside, and rutin (Xu et al., 2007) exhibited free radical scavenging activity. Furthermore, epigallocatechin and quercetin 3-(4′-acetylrhamnoside) 7-rhamnoside were identified (Han N. et al., 2023).

Lignans metabolites in *P. urinaria* have been extensively researched, with a particular focus on one dominant species of the metabolites. Fang et al. (Fang et al., 2008) utilized NMR and ESI-MS techniques to identify nine anti-inflammatory and antioxidant metabolites in *P. urinaria* ethanol extract. Chang et al. (Chang et al., 2003) also conducted a similar analysis on lignans from *P. urinaria*, identifying known metabolites like phyllanthin, niranthin, phyltetralin, hypophyllanthin, nirtetralin, lintetralin, isolintetralin, heliobuphthalmin lactone, and virgatusin (Mao et al., 2016), as well as newly discovered 5-Demethoxyniranthin, urinatetralin, dextrobursehernin, and urinaligran. In addition, Han et al. (Han N. et al., 2023) isolated dihydrocubebin, lyoniresiol, (7S, 7′S, 8R, 8′R)-icariol A2, evofolin B, 4-oxopinoresinol, syringaresinol, episyringaresinol from *P. urinaria* extract. Boehmenan and repandusinic acid A are extracts from the roots, stems, and leaves of *P. urinaria*, known for their inhibitory effects on α-glucosidase. Using HPLC-SPE-NMR, seven structurally similar lignans were analyzed from the methanol extract of *P. urinaria* (Wang and Lee, 2005), with niranthin, phyllanthin, and phyltetralin being the main metabolites identified, alongside hypophyllanthin, virgatusin, nirtetralin, and lintetralin. Fan et al. (Fan et al., 2015) developed a precise and rapid HPLC-MS/MS method for the specific identification of four lignans found in rat plasma after oral administration of *P. urinaria* extract, which includes niranthin, nirtetralin, hypophyllanthin, and phyllanthin, this method shows potential for qualitative analysis of other lignans.

#### 3.4.3 Inactivate HSCs and inhibit HSCs proliferation

The activation of HSCs is influenced by changes in the cell microenvironment, leading to metabolic reprogramming, increased collagen synthesis, decreased apoptosis, delayed senescence, and reduced immune clearance. These factors collectively contribute to the progression of liver fibrosis. In liver fibrosis diseases, the upregulation of lysyl oxidases (LOX), tissue inhibitor of metalloproteinase 1 (TIMP1) and α-smooth muscle actin (α-SMA) are observed alongside HSCs activation (Cai et al., 2020). Abnormal activation of signaling pathways such as TGF-β/Smad, Wnt/β-catenin, Notch, PI3K/Akt, Hedgehog, Hippo/YAP, MAPK, and Toll-like receptor 4 (TLR4) can further promote HSCs activation. Studies suggest that reducing levels of α-SMA, CTGF, PDGFA/B, VEGFA, IL-11, IL-6, and CXCL12 may help inactivate HSCs (Dou et al., 2018). Flavonoids, terpenoids, alkaloids, and other plant metabolites offer a natural approach with multi-target, multi-level, and multi-channel advantages to regulate related proteins and potentially reverse liver fibrosis (Zheng et al., 2023). Natural products provide advantages such as easy accessibility, potent pharmacological effects, and minimal side effects, showing promising therapeutic potential in the treatment of liver fibrosis.

Phenolic acids and flavonoids have been shown to possess anti-liver fibrosis properties by regulating hepatocyte apoptosis through specific signaling pathways (Xu et al., 2023). Kumaran et al. (Kumaran and Joel Karunakaran, 2007) conducted research demonstrating the antioxidant activity of five *Phyllanthus* plant species, with *P. urinaria* ranking second. Quercetin, known for its strong antioxidant and anti-inflammatory properties (Shen et al., 2008), has been found to inhibit HSCs activation by affecting the NF-κB/MAPK and Bcl-2/BAX pathways, thereby delaying the progression of liver fibrosis. In an induced liver fibrosis model in Sprague Dawley (SD) rats, treatment with quercetin resulted in reduced levels of serum markers, improved liver appearance, decreased collagen deposition, downregulated NF-κB/p38, and MAPK/BAX expression, increased Bcl-2 levels, and reduced marker of HSCs activation and inflammatory factors (Wang et al., 2017). Liposomal modification of quercetin has been shown to improve liver fibrosis induced by concanavalin A in mice. Treatment with liposomal quercetin led to enhanced collagen deposition in the liver, inhibition of HSCs activation, reduced expression of α-SMA, NF-κB, and TGF-β (Wan et al., 2014). Li et al. (Li et al., 2016) confirmed the effectiveness of quercetin in improving liver fibrosis through both *in vivo* and *in vitro* experiments. Using a mouse liver fibrosis model induced by intraperitoneal injection of CCL4, it was observed that oral quercetin treatment resulted in lower portal inflammation scores, decreased serum enzymes ALT and AST, as well as reduced collagen deposition and fibrosis scores. *In vitro* experiments demonstrated that quercetin treatment led to decreased expression of TGF-β1, α-SMA, HMGB1, TLR2/TLR4, and NF-κB p65 proteins in COL1α1 mRNA, suggesting that quercetin may inhibit HSCs activation and improve liver fibrosis by modulating the HMGB1/TLR/NF-κB signaling pathway. Aslam et al. (Aslam et al., 2022) also showed that quercetin has the potential to ameliorate liver fibrosis by antagonizing the hedgehog pathway. Their study induced liver fibrosis in SD rats using thioacetamide (TAA) and observed various indicators post-quercetin treatment. The results indicated an increase in serum SOD levels and a decrease in the expression of hedgehog pathway markers Shh, Ihh, Ptch-1, Smo, and Gli3, as well as inflammation-related markers TNF-α and NF-κB. This suggests that quercetin may regulate the hedgehog pathway and oxidative stress to improve liver fibrosis. Additionally, Hernandez-Ortega et al. (Hernandez-Ortega et al., 2012) found that quercetin reduced AST, ALT, and GGT levels in mice with liver fibrosis, decreased the area of liver fibrosis and collagen deposition, increased SOD, CAT serum enzymes and mRNA levels, inhibited inflammation by reducing the expression of markers TNF-α, IL-6, TGF-β1, COL1α1, CTGF, TIMP-1, and α-SMA, induced HSCs apoptosis, and increased the expression of anti-liver fibrosis molecules TGF-β1, MMP2, and MMP9. Quercetin has also been shown to combat liver fibrosis by modulating macrophage activation and polarization in both *in vitro* and *in vivo* experiments (Li et al., 2018). *In vivo* studies have shown that treatment with quercetin significantly improves necrotic inflammation damage and liver collagen deposition induced by CCL4. Quercetin also inhibits the expression of α-SMA, a marker of HSCs activation, reduces protein levels of desmin and vimentin, downregulates macrophage markers F4/80 and CD68, decreases mRNA expression of TNF-α, IL-1β, IL-6, and MCP-1, and reduces macrophage recruitment. Additionally, *In vitro* experiments have revealed that quercetin inhibits the polarization of M1 macrophages in RAW264.7 cells, resulting in reduced levels of TNF-α, IL-1β, IL-6, and NOS2, as well as decreased expression of Notch1. Another flavonoid, kaempferol found in *P. urinaria*, has also shown potential in reversing liver fibrosis (Zhou et al., 2022). In a mouse model of CCL4-induced liver fibrosis, treatment with kaempferol led to improved collagen deposition and reduced expression of α-SMA and COL1α1. The mechanism underlying the inhibition of HSCs differentiation may involve the downregulation of Notch/Jag1 and the upregulation of miR-26b-5p expression. Xu et al. (Xu et al., 2019) investigated the therapeutic effects of kaempferol on mice with CCL4-induced liver fibrosis. The study demonstrated that kaempferol led to significant repair of liver damage, reduction in serum levels of HA, LN, AST, and ALT, decreased collagen deposition, and downregulation of COL1α1 and α-SMA RNA and protein expression. *In vitro* experiments suggested that kaempferol could potentially reduce the elevation of Smad2 and Smad3 induced by TGF-β1 by selectively binding to the ATP binding site of ALK5. Besides, gallic acid was found to possess anti-inflammatory and antioxidant properties. El-Lakkany et al. (El-Lakkany et al., 2019) confirmed through *in vivo* and *in vitro* experiments that gallic acid effectively regulates the proliferation and activation of HSCs for the treatment of liver fibrosis. Gallic acid exhibited the ability to inhibit HSCs cell proliferation in a dose-dependent and time-dependent manner, decrease α-SMA expression, lower the levels of liver injury markers such as PCNA, PDGF-BB, TIMP-1, HP, and reduce collagen deposition in a rat model of liver fibrosis.

#### 3.4.4 ECM degradation and remodeling

ECM degradation and remodeling are crucial processes in the dynamic formation of liver fibers. Matrix metalloproteinases (MMPs), including collagenases (MMP1, MMP8, MMP13) and gelatinases (MMP2, MMP9), play a significant role in ECM degradation, with MMP12 also contributing. Elastase is essential for activating MMPs, maintaining a balance between MMP/TIMP levels and VEGF-mediated angiogenesis is vital for improving fibrosis and tissue remodeling (Rockey et al., 2015). The pathophysiological characteristics of liver cirrhosis involve liver damage and fibrosis. Treatment approaches for liver cirrhosis encompass the management of hepatitis infections, reduction of cirrhosis risk factors, and prevention of liver damage from harmful substances (Ge and Runyon, 2016). *Phyllanthus urinaria* L. exhibits hepatoprotective potential (Srirama et al., 2012) and may offer benefits in addressing liver damage conditions (Sharma et al., 2011). Existing literature suggests that *P. urinaria* can inhibit the cytochrome P450 enzyme, thus reducing liver toxicity and providing protective effects to the liver. Ellagic acid and quercetin, identified as the metabolites in *P. urinaria*, have demonstrated effective protection of rat livers from damage induced by TAA (Lee et al., 2014).

Research findings suggest that ellagic acid may regulate TAA-induced hepatitis by modulating the mitogen-activated protein kinase through the TLR/MAPK/NF-κB signaling pathway. *In vivo* experiments on TAA model mice showed elevated levels of serum enzymes ALT, AST, and ALP, indicative of liver damage. Additionally, increased MDA levels and decreased TAC and GPX levels pointed to lipid peroxidation, while upregulation of MMP2 and MMP9 indicated liver fibrosis progression. However, treatment with ellagic acid and quercetin resulted in decreased ALT, AST, and ALP levels, reduced MDA levels by 34.8% and 54.65%, significantly increased TAC and GPX levels, and decreased expression of MMP2 and MMP9. These results suggest the potential therapeutic use of ellagic acid and quercetin in mitigating liver fibrosis (Afifi et al., 2018). CCL4 has been demonstrated to induce liver damage in rats, while *P. urinaria* has been shown to have a liver-protective effect. In a study by Lee et al. (Lee et al., 2006), treatment with the methanol extract of *P. urinaria* resulted in decreased levels of GOT and GPT enzymes in liver fibrosis model rats. Additionally, the methanol extract of *P. urinaria* led to increased levels of SOD, GSH, and GPX, ultimately reducing liver tissue necrosis. Four lignans, namely, hypophyllanthin, phyllanthin, nirtetralin, and niranthin, have been identified as quality control markers for *P. urinaria* extract. Guo and colleagues (Guo et al., 2017) used metabolomics to investigate the protective mechanism of *P. urinaria* against CCL4-induced liver damage. Their findings suggest that *P. urinaria* can alleviate branched-chain amino acid catabolism disorders and enhance free radical scavenging activity by modulating L-carnitine, taurocholic acid, and amino acid metabolism to ameliorate liver damage.

Phyllanthin, a lignan metabolite found in *P. urinaria* extract, plays a crucial role in regulating the innate immune response of phagocytes, scavenging free radicals, acting as an antioxidant, and protecting the liver (Yuandani et al., 2013). Studies have also shown that phyllanthin can protect against liver damage by modulating reactive oxygen species (ROS) (Chirdchupunseree and Pramyothin, 2010) and reducing lipid peroxidation to alleviate ethanol-induced liver damage (Krithika et al., 2009). In a study by Krithika et al. (Krithika et al., 2016), female mice were orally administered CCL4 to induce a liver fibrosis model. Treatment with phyllanthin was found to improve liver fibrosis by reducing the levels of TNF-α, NF-κB, and TGF-β1 in liver tissue. Additionally, it decreased the levels of LPO, ALT, and AST, as well as the mRNA expression of liver fibrosis markers such as fibronectin, TGF-β1, and COL1α1. Moreover, phyllanthin decreased the protein expression of TNF-α and NF-κB p65, and reduced collagen deposition to counteract ECM synthesis.

Encapsulating phyllanthin in nanoparticles using polylactic-co-glycolic acid (PLGA) enhanced its bioavailability and mitigated liver fibrosis by reducing collagen deposition (Krithika et al., 2019). Phyllanthin nanoparticles, prepared with PLGA, acetone, and Tween via the nanoprecipitation method, were administered to a CCL4-induced liver fibrosis model. The results showed that treatment with phyllanthin nanoparticles resulted in decreased ALT and AST levels, repair of cell membrane damage from CCL4, and improvement in liver fibrosis. Krithika et al. (Krithika et al., 2015) employed molecular docking to identify the specific binding site of phyllanthin and ALK5, followed by *in vivo* experiments to validate the anti-fibrosis mechanism of phyllanthin. The molecular docking results indicated a strong binding affinity between phyllanthin and ALK5. After phyllanthin treatment, ALT, AST, and liver collagen levels decreased, reversing pathological liver tissue changes induced by CCL4. Furthermore, the expression of TGF-β1, ALK5, p-Smad2, and p-Smad3 decreased played a crucial role in ECM remodeling.

Huangjia Ruangan Granule (HJRG) is a compound preparation of TCM known for its anti-liver fibrosis properties (Cai et al., 2022). It comprises various metabolites such as *P. urinaria*, Hedysarum Multijugum Maxim (Huangqi), Trionyx sinensis Wiegmann (Biejia), Radix Puerariae (Gegen), Radix Bupleuri (Chaihu), Ganoderma (Lingzhi), Radix Paeoniae Rubra (Chishao), Radix Salviae (Danshen), Panax Notoginseng (Sanqi), and Abri Herba (Jigucao). Through an HPLC test conducted on a CCL4-induced liver fibrosis model in SD rats, the presence of gallic acid, puerarin, tanshinone IIA, and other metabolites was confirmed. Treatment with HJRG led to improvements in liver tissue necrosis, inflammation, and fatty degeneration in the rats. There were significant reductions in parameters such as fiber intervals, severity scores, and collagen fiber-positive areas. Moreover, the levels of AST and ALT enzymes decreased, along with PCI, COL IV, LN, HA, and α-SMA, suggesting that HJRG has the potential to alleviate liver damage and fibrosis. The mechanism behind this effect is likely linked to HJRG’s ability to reduce oxidative stress and inflammation, possibly through modulation of the TNF-α/MAPK and NF-κB signaling pathways. Corilagin, a vital metabolite found in *P. urinaria* extract, has demonstrated efficacy in treating liver fibrosis/cirrhosis caused by schistosomiasis. This therapeutic effect is attributed to its modulation of the IL-13 signaling pathway and suppression of GATA3 to regulate the Th1/Th2 balance (Huang et al., 2013). Moreover, corilagin displays hepatoprotective properties by reversing elevated levels of AST and ALT enzymes induced by liver injury, reducing liver MPO activity, and increasing inflammatory markers like CINC-1, CINC-3, ICAM-1, IL-6, and TNF-α. The administration of corilagin can counter these alterations, potentially through the inhibition of the PI3K/Akt signaling pathway (Liu et al., 2017).

#### 3.4.5 Clinical applications

Clinical trials have demonstrated that anti-viral therapy can significantly enhance the clinical outcomes of liver fibrosis/cirrhosis by eradicating or managing the disease-causing factors (Wang et al., 2022). While nucleotide analogs alone may have limited efficacy in reversing liver fibrosis/cirrhosis induced by hepatitis, the addition of TCM can be a viable complementary treatment approach (Zhang and Schuppan, 2014). In a multi-center clinical controlled trial led by Rong et al. (Rong et al., 2022), the therapeutic benefits of Biejia Ruangan Tablets combined with nucleoside analogs were assessed in patients with CHB related liver fibrosis, revealing a higher rate of fibrosis/cirrhosis regression in those treated with Biejia Ruangan Tablets. The combination of Fuzheng Huayu (FZHY) - comprising Radix Salvia Miltiorrhizae (Danshen), Pollen Pini (Songhuafen), Semen Persicae (Taoren), Gynostemma Pentaphyllammak (Jiaogulan), Cordyceps (Chongcao) - and entecavir (ETV) has been proven to ameliorate liver fibrosis by collagen degradation without inducing hepatotoxicity (Gui et al., 2020). In a study by Xing et al. (Xing et al., 2023), a multi-center randomized controlled clinical trial was conducted to evaluate the efficacy of Ruangan Granules (*P. urinaria*, Trionycis Carapax, Notoginseng Radix et Rhizoma, Salviae Miltiorrhizae Radix et Rhizoma, Persicae Semen, Astragali Radix, Atractylodis Macrocephalae Rhizoma, Poria, Curcumae Radix, Aurantii Fructus, Lobeliae Chinensis Herba, Schisandrae Chinensis Fructus) in patients with CHB related liver fibrosis. The study included 240 patients with severe liver fibrosis/cirrhosis and CHB, who were treated with Ruangan Granules in addition to anti-HBV drugs for 48 weeks. Results indicated that the group receiving Ruangan Granules showed improved liver pathology, reduced fibrous tissue proliferation, lower Knodell HAI score, Ishak score, and ultrasound semi-quantitative score, liver stiffness measurement (LSM), suggesting an improvement in liver fibrosis. Furthermore, there was an increase in HBV DNA negative conversion rate, ALB levels, and a decrease in ALT and AST levels. Follow-up after 2 years revealed a lower incidence of HCC in the Ruangan Granule group with no significant adverse events, indicating a favorable safety profile of the drug.

### 3.5 Application status of *P. urinaria* in anti-HCC

#### 3.5.1 The epidemiology and treatment of HCC

Primary liver cancers, particularly HCC, are a significant global public health concern, ranking sixth in incidence and third in mortality among tumors (Bray et al., 2024). Treatment approaches for HCC are determined by factors such as liver function, tumor burden, and cancer stage. Options include curative therapies like liver transplantation, resection, transarterial chemoembolization, and radiofrequency ablation, often used in combination with early-stage cases (Torimura and Iwamoto, 2022). Non-definitive therapies for HCC include first-line systemic treatments such as transarterial radioembolization, transarterial chemoembolization, antibody therapies, immune checkpoint inhibitors, and molecularly targeted agents. However, due to the insidious nature of HCC onset and constraints in diagnostic capabilities, only approximately 20% of patients qualify for curative treatment. The majority of HCC cases, over 80%, are detected at an advanced stage, precluding the opportunity for curative interventions (Zhou et al., 2023). The use of botanical drugs or natural products derived from botanical drugs in the prevention and treatment of HCC provides a complementary alternative therapy. Natural products have advantages in the treatment of HCC by reducing the side effects linked to radiotherapy and chemotherapy, improving long-term effectiveness, and catering to patients who are not suitable for surgery, radiotherapy, or chemotherapy. These treatments can assist in tumor control, symptom relief, and enhancing quality of life. Additionally, they can enhance the body’s immune response, reduce metastasis and postoperative recurrence, and potentially prolong the survival of some patients with tumors. Several studies have demonstrated the potential anticancer properties of *P. urinaria*. For example, it has been found that the aqueous extract of *P. urinaria* can induce apoptosis in HL-60 cells through the ceramide-related pathway, leading to upregulation of caspase 3 and BAX expression while downregulation of the expression of Bcl-2, Fas receptor, and Fas ligand genes, thus showing promise in treating human myelogenous leukemia (Huang et al., 2004a). Additionally, research has shown that *P. urinaria* extract can induce apoptosis in Lewis cells and combat lung cancer through the mitochondria-related intrinsic pathway, with upregulation of caspase 3 expression and downregulation of Bcl-2 expression, all while demonstrating minimal toxicity to normal cells (Huang et al., 2003). Supplementary Table S4 summarizes the pharmacological activities of *P. urinaria* based on extraction methods, mechanisms applied, and effects.

#### 3.5.2 The metabolites of anti-HCC

The anti-HCC metabolites of *P. urinaria* are highlighted in Supplementary Table S1. Utilizing a combination of tannin ultrasonic-assisted extraction (UAE) and response surface methodology (RSM) proves advantageous for the extraction and characterization of phenolic metabolites from *P. urinaria* extract (Liu et al., 2018). He et al. (He et al., 2022) successfully identified 15 phenolic metabolites through spectral analysis, 1D-NMR, and 2D-NMR techniques, including furosin, geraniin, corilagin, repandinin B, repandusinic acid A, and mallotinin. Corilagin, a polyphenolic metabolite derived from *P. urinaria*, has demonstrated anti-atherosclerotic properties both *in vitro* and *in vivo*. Jikai et al. (Jikai et al., 2002) efficiently separated corilagin and gallic acid in the methanol extract of *P. urinaria* using high-speed countercurrent chromatography (HSCCC). Corilagin was successfully isolated from the methanol extract of *P. urinaria* with a yield of 25 mg/g. Moreover, dehydrochebulic acid trimethyl ester was also identified (Hu et al., 2014). The acetone extract of *P. urinaria* contains geraniin and 1,3,4,6-tetra-O-galloyl-β-D-glucose (Zhong et al., 2013), which have exhibited inhibitory effects on the growth of HSV-1 and HSV-2 (Yang et al., 2007a). Additionally, gemin D and hippomanin A have shown anti-HSV-2 effects (Yang et al., 2007b). Geraniin, identified through NMR spectrum and mass spectrometry of the *P. urinaria* acetone extract, is a hydrolyzable tannin with antioxidant properties (Lin et al., 2008). Phyllanthusiin A and isostrictinin, both polyphenols, are also present in the *P. urinaria* extract (Han N. et al., 2023). Various terpenes (Hu et al., 2014), including triterpenes like glochidiol and oleanolic acid, diterpenes such as cleistanthol and spruceanol, sesquiterpenes like cloven-2β,9α-dio, and monoterpenes such as (6R)-menthiafolic acid, have been identified. Wu et al. (Wu et al., 2017) identified several triterpenoids in the ethanol extract of *P. urinaria*, including betulin, β-betulinic acid, 3-oxo-friedelan-28-oic acid, oleanolic acid, 28-norlup-20(29)-ene-3β, 17β-diol, and 3R-Z-coumaroyltaraxerol. These metabolites have demonstrated the ability to inhibit the production of NO in mouse macrophages, suggesting potential anti-inflammatory properties. Other metabolites found in *P. urinaria* extracts included montanoic acid ethyl, methyl brevifolin carboxylate (Calixto et al., 1998), sterols such as β-Sitosterol-3-O-β-D-glucopyranoside and β-Sitosterol (Fang et al., 2008), alkaloids like phyllurine (Ueda and Yamamura, 2000) and phyllanthurinolactone (Ueda et al., 1998), as well as 5-hydroxymethyl-2-furaldehyde and epigallocatechin-(4β->8)-catechin (Han N. et al., 2023). Furthermore, excoecarianin (Cheng et al., 2011) was identified in the acetone extract of *P. urinaria* and showed specific activity against HSV-2 virus infection. Phthalic acid bisester (Satyan et al., 1995) was also isolated from the petroleum ether extract of *P. urinaria*.

#### 3.5.3 Inhibition of tumor proliferation

External factors, such as infection, can influence the inhibition of tumor proliferation. HBV infection has the potential to activate cancer-related signaling pathways and modify the immune microenvironment by triggering host gene instability and epigenetic remodeling through viral integration. This process can ultimately facilitate the development of HCC (Jiang et al., 2021). Flavonoids and tannins demonstrate cytotoxic effects that hinder tumor cell proliferation, prompt tumor cell cycle arrest, and curb HCC growth. Quercetin and corilagin have been recognized as metabolites with anti-HCC properties (Teodor et al., 2020b). Flavonoids can boost tumor cell apoptosis by up-regulating oncogenes, down-regulating pro-oncogenic genes, and modulating various signaling pathways like Wnt/β-catenin, MAPK, AP-1, and NF-κB. Moreover, they influence enzymes such as PTK, PKC, XO, iNOS, and COX-2 to impede tumor cell proliferation and regulate the cell cycle across different phases (S/G2, G1, S, and G2) (Hazafa et al., 2020).

Sorafenib, a chemotherapy drug used for treating HCC, may lose effectiveness over time due to acquired resistance. Research indicates that quercetin shows promise in reversing this acquired resistance to sorafenib. In a study conducted by Zhang et al. (Zhang et al., 2024), a drug-resistant HCC cell line named Huh7^R^ was established. *In vitro* experiments demonstrated that quercetin could maintain cell morphology in both Huh7 and Huh7^R^ cell lines and decrease the number of HCC cell clones, indicating its inhibitory effect on HCC cell growth. The analysis suggests that the mechanism behind this reversal of drug resistance may involve the protein kinase B signaling pathway and the EGFR signaling pathway. Molecular docking studies revealed a strong binding affinity between quercetin and Akt1. Treatment with quercetin led to decreased expression of p-EGFR/EGFR, p-Akt/Akt, p-ERK/ERK, and Bcl-2 proteins, while increasing Cleaved PARP1 and BAX expression. In a drug-resistant tumor model in nude mice, quercetin treatment significantly reduced tumor weight and volume, with protein expression patterns consistent with *in vitro* findings. Corilagin has been utilized in clinical studies, demonstrating no acute or subacute toxicity following safety assessment (Wang et al., 2023). *In vitro* experiments have demonstrated that corilagin inhibits the proliferation of HCC cells (Bel7402, SMMC7721) in a concentration-dependent manner. It causes cell cycle arrest in the G2/M phase, leading to the downregulation of key proteins cyclin B1 and ccdc2 in the G2/M phase, while also enhancing the inhibition of oncogenes p21, Cip1, and p-p53. Furthermore, *in vivo* studies using the Balb/c nude mouse xenograft model with MHCC97-H cells have shown that treatment with varying doses of corilagin (10, 20, or 30 mg/kg) results in a significant reduction in tumor mass and volume, demonstrating a dose-dependent inhibition of tumor growth. (Ming et al., 2013). Furthermore, corilagin has exhibited the ability to suppress the growth of liver tumors in athymic nude mice injected with Hep3B cells, with 15 mg/kg of corilagin leading to a notable decrease in tumor growth and volume compared to the control group. Liver function tests and blood markers AST and ALT have remained within normal ranges, indicating no observable toxicity associated with corilagin treatment (Hau et al., 2010).

#### 3.5.4 Anti-tumor metastasis, invasion, and angiogenesis

Tumor metastasis and spread are the primary causes of mortality in cancer patients, closely associated with EMT and mesenchymal-to-epithelial transition (MET). These transitions enable tumor cells to escape the primary site and disseminate through the bloodstream to form new tumors nearby or in distant locations (Jassim et al., 2023). The activation of invasion and metastasis is a fundamental characteristic of cancer, an effective strategy to combat tumor metastasis involves combining therapies targeting ECM adhesion molecules (de Visser and Joyce, 2023), intra-tumor interactions, EMT, and protease release (Fares et al., 2020). Given the highly vascular nature of HCC, the formation of blood vessels is essential for tumor growth, aiding in tumor cell invasion and metastasis. Consequently, inhibiting vascular endothelial growth factor receptor (VEGFR) through anti-angiogenesis therapy is a primary approach to prevent HCC invasion (Liu et al., 2022). Studies have shown that *P. urinaria* extract can hinder tumor growth by targeting the extracellular signal-related kinase 1/2 (ERK1/2) signaling pathway and angiogenesis (Tseng et al., 2012). Upon treatment with *P. urinaria* extract, there is a decrease in the protein expression of ELk-1, c-Raf, c-Myc, c-Jun/AP-1, Pan-Ras, HIF-1α, VEGF, and iNOS (Lee et al., 2016). In a study by Huang et al. (Huang et al., 2024), it was discovered that reducing levels of MMP 2 and MMP 9 in the MAPK family can impede tumor invasion and metastasis by targeting the ERK/JNK and hypoxia pathways. Quercetin has been shown to regulate SMURF2/RhoC, resulting in the inhibition of HCC invasion and metastasis. Experimental data have shown that RhoC protein and mRNA levels were elevated in highly metastatic and invasive HCC cell lines, but quercetin treatment reduced RhoC levels in a concentration-dependent manner, thereby inhibiting the invasion and migration abilities of HCC cells. Further analysis revealed that quercetin inhibits invasion and migration by suppressing RhoC expression through the protease pathway. In various mouse models, including those with subcutaneous transplanted tumors, orthotopic tumors, and patient tumor metastasis, quercetin treatment led to reduced tumor mass and volume, along with inhibition of tumor lung metastasis. Immunohistochemical staining indicated increased SMURF2 expression, suggesting that quercetin may hinder HCC invasion and metastasis by reducing RhoC expression via SMURF2 and the ubiquitination pathway. Inhibition of protease activity has been shown to suppress tumor metastasis, with studies demonstrating that targeting the PI3K/Akt and JNK1/2 signaling pathways reduces MMP 2, MMP 9, urokinase plasminogen activator (u-PA), NF-κB, and AP-1 mRNA and protein expression in tumor cells, thereby impeding tumor invasion and metastasis. Tang et al. (Tang et al., 2015) and Huang et al. (Huang et al., 2006) have both confirmed that *P. urinaria* inhibits tumor invasion and angiogenesis by suppressing MMP activity.

TCM formulations with spleen-strengthening and detoxification properties have shown promise in improving liver function and quality of life in HCC patients. The Yiqi Jianpi Jiedu (YQJPJD) prescription, containing botanical drugs like *P. urinaria*, was specifically developed for this purpose. Wu et al. (Wu et al., 2022) conducted a study to elucidate the mechanism of action of the YQJPJD prescription in treating HCC using network pharmacology and *in vitro* experiments. Their findings highlighted the role of quercetin and other active metabolites in the prescription in HCC treatment. Pathway analysis revealed associations with cancer and hepatitis B signaling pathways. Protein-protein interaction analysis identified key target proteins like TP53, Akt1, STAT3, and MAPK. Molecular docking results indicated strong binding of active metabolites to proteins such as MAPK3, RAC1, and β-catenin. *In vitro* experiments demonstrated that YQJPJD treatment led to a concentration-dependent increase in wound healing area in HepG2 and Hep3B cells. Transwell migration experiments confirmed the ability of YQJPJD to inhibit the invasion and migration of HCC cells. Moreover, YQJPJD was found to suppress the activation of the PI3K/Akt pathway, as evidenced by reduced levels of p-PI3K and p-Akt in treated cells, suggesting a potential mechanism for its anti-invasive effects. Studies have also shown that *Phyllanthus Urinaria* L. Anti-neoplastic Decoction (PAD) has the potential to suppress invasion and metastasis of HCC. Wei et al. (Wei et al., 2024) discovered that PAD, a key botanical drug in ‘yiqi jianpi jiedu huayu’, can combat HCC by targeting the PI3K/Akt pathway. The primary metabolites of PAD, such as quercetin and kaempferol, play a crucial role in this process. *In vitro* experiments showed that PAD effectively reduced levels of p-PI3K, Akt, and p-Akt proteins in Hep3B and HepG2 cells.

Huang et al. (Huang et al., 2021) investigated the TCM compound CP, consisting of *P. urinaria*, Astragalus mongholicus Bunge, Curcuma aromatica Salisb., Scutellaria barbata D., and Cremastra appendiculata Makino, commonly used in treating HBV-related HCC. Their study delved into the inhibitory mechanism of this metabolite on HCC through a combination of *in vitro* and *in vivo* experiments, along with bioinformatics analysis. Network pharmacology results revealed the pivotal pathways and targets of *P. urinaria* in HCC, namely, the Wnt/β-catenin signaling pathway and Cav-1 protein. In their *in vitro* experiments, the researchers utilized HepG2 cells infected with HBx-expressing lentiviral particles to establish HepG2-HBx cells, which displayed heightened migration and invasion capabilities compared to HepG2 cells. Treatment with *P. urinaria* attenuated the wound-healing ability of HCC cells and reduced cell migration through the transwell chamber. Western blot experiments demonstrated that the compound downregulation the expression of EMT marker proteins vimentin and N-cadherin, while upregulation E-cadherin expression. Subsequent investigations have revealed that *P. urinaria* exerts inhibitory effects on the Akt/GSK-3β/β-catenin pathway, thereby suppressing EMT, indicating its potential to impede the migration and invasion of HBV-related HCC cells. Utilizing an *in vivo* metastatic zebrafish xenograft model, the researchers demonstrated the ability of *P. urinaria* treatment to restrain tumor dissemination in zebrafish. The combined findings from both *in vitro* and *in vivo* studies substantiate the efficacy of *P. urinaria* in inhibiting HCC metastasis.

Exosomes and autophagy are critical factors in shaping the tumor microenvironment, and overcoming resistance to lenvatinib is a promising approach to improving treatment outcomes in HCC. In a recent study by Liao et al. (Liao et al., 2024), the effectiveness of a combination of lenvatinib and TCM compound CP was investigated in the context of HBV-related HCC. The results showed that the combination therapy had superior therapeutic effects compared to lenvatinib alone, leading to enhanced inhibition of cell proliferation in HepG2 cells. Moreover, *in vivo* experiments demonstrated a significant reduction in subcutaneous tumor mass and volume in mice treated with CP in combination with lenvatinib. Transcriptomic sequencing analysis revealed that this combined treatment altered the expression of exosomal miRNAs in HepG2 cells, specifically upregulating miR-181b-5p and miR-423-5p. PCR verification confirmed an increase in the expression of tumor suppressor genes like miR-193-3p and a decrease in proto-oncogenes such as miR-7704 following the combination therapy. Gene enrichment analysis indicated the involvement of differentially expressed miRNAs in the autophagy pathway, while Western blot experiments showed increased levels of autophagy-related proteins beclin-1 and LC3-II, and a decrease in p62 expression. These findings suggest that *P. urinaria* has the potential to enhance the efficacy of chemotherapy drugs, thereby inhibiting invasion and metastasis in HCC.

#### 3.5.5 Inducing tumor apoptosis

Induction of tumor apoptosis is a highly regulated cellular response that involves detection of extracellular signals, amplification of local signals, and integration of cellular information into the central processing unit for cell death. *Phyllanthus urinaria* has been investigated for its ability to trigger apoptosis (Huang et al., 2010) in various tumor cells through mechanisms such as loss of mitochondrial membrane potential (Wu et al., 2012), activation of the Fas receptor (Huang et al., 2004a)/ligand signaling pathway (Tang et al., 2010), and inhibition of telomerase enzyme activity (Huang et al., 2009). Mitochondria play a critical role in HCC metabolism, influencing processes like mitophagy and apoptosis through the regulation of mtDNA. Targeting Mdivi-1, BAPTA-AM, and microRNAs to suppress mitochondrial fission and induce apoptosis in HCC has demonstrated potential (Zhang et al., 2020).

Chudapongse et al. (Chudapongse et al., 2010) conducted research demonstrating that *P. urinaria* has the ability to regulate mitochondrial metabolism and induce apoptosis in HCC cells. The methanol extract of *P. urinaria* was found to contain 354 ± 27 mg/g of gallic acid, with experiments revealing a concentration-dependent inhibition of HepG2 cell proliferation and alterations in cell morphology. Treatment with *P. urinaria* was also observed to suppress mitochondrial oxidative phosphorylation, decrease intracellular ATP levels, modulate Ca^2+^ levels, trigger mitochondrial dysfunction, and ultimately result in cell apoptosis. Corilagin, a polyphenolic metabolite present in *P. urinaria*, is identified as one of its primary metabolites. Deng et al. (Deng et al., 2018) reported that corilagin can induce apoptosis in HCC cells through both the death receptor pathway and the mitochondrial apoptosis pathway. MTT assay demonstrated that corilagin inhibited the viability of various HCC cells (MHCC97-H, Bel-7402, and SMMC-7721) in a concentration-dependent manner, leading to morphological changes like nuclear fragmentation and chromatin condensation. These findings suggest that corilagin possesses the capability to impede the proliferation of HCC. Flow cytometry analysis confirmed that following corilagin treatment, there was an increase in the proportion of apoptosis in HCC cells, a decrease in the mitochondrial membrane potential ratio, elevation in apoptosis-related proteins such as Cyto c, caspase 8, and TP53, reduction in p-Akt and Bcl-2 protein expression, and cleavage of caspase 3, caspase 9, and PPAR.

Lignans, metabolites of *P. urinaria*, exhibit inhibitory effects on HepG2 cells (Teodor et al., 2020a). The proto-oncogenes c-myc and Bcl-2 are crucial in apoptosis. Giridharan et al. (Giridharan et al., 2002) isolated lignan metabolites from *P. urinaria* extract, identifying them as 7′-hydroxy-3′,4,5,9,9′-pentamethoxy-3,4-methylene dioxy. These metabolites upregulate c-myc, downregulation Bcl-2, activate caspase, and inhibit telomerase activity, inducing HepG2 cell apoptosis *in vitro*. Kaempferol, another metabolite from *P. urinaria*, triggers HepG2 cell apoptosis via the endoplasmic reticulum stress/CHOP pathway. It inhibits HepG2 activity depending on concentration and time, leading to increased LDH activity, upregulation of GRP94 and GRP78 endoplasmic reticulum stress markers at mRNA and protein levels, and induction of CHOP and caspase 3 apoptosis proteins in HCC cells (Guo et al., 2016). Furthermore, an aqueous extract of *P. urinaria* induces apoptosis in HepG2 cells in a concentration-dependent manner, displaying typical apoptotic characteristics like apoptotic bodies, cell shrinkage, membrane blebbing, and gelation. DNA fragmentation was evident through electrophoresis. Importantly, this extract shows no significant toxicity to normal liver cells WRL68 and endothelial cells HUVEC, indicating good safety (Huang et al., 2004b).

#### 3.5.6 Modulate signaling pathways and immune microenvironment

Regulating cell transduction pathways and immune responses are crucial strategies in cancer treatment. Research has shown that *P. urinaria* can effectively influence various signaling pathways implicated in tumorigenesis (Tang et al., 2013), such as NF-κB, PI3K/Akt, and ERK/JNK/MAPK pathways, to combat HCC (Saahene et al., 2021). Jantan et al. (Jantan et al., 2019) conducted a review on the regulatory properties of *P. urinaria* and its metabolites, such as ellagic acid, quercetin, and corilagin on innate and adaptive immunity, suggesting its potential in HCC treatment through immune modulation. Diethylnitrosamine (DEN) is a well-known hepatotoxic carcinogen that reduces 8-OHdG levels, inhibits liver protein synthesis, generates free radicals, and induces lipid peroxidation. It is commonly used to establish animal models for HCC.

Previous studies (You et al., 2021) have shown that phyllanthin induces apoptosis through the PI3K/Akt/mTOR signaling pathway, providing resistance against HCC. Treatment with phyllanthin in HCC rat models induced by DEN led to a decrease in tumor count and incidence. Moreover, markers such as CEA, AFP, 8-OHdG, LDH, GGT, ALT, AST, and ALP showed elevated levels. Additionally, the mRNA expression of TP53, caspase 3, caspase 9, and BAX increased, while Bcl-2, mTOR, PI3K, and Akt expression decreased, phyllanthin treatment also induced apoptosis in HepG2 cells *in vitro*. Another study by Seydi et al. (Seydi et al., 2018) demonstrated that kaempferol can modulate ROS signaling pathways in HCC treatment. In an animal model induced with DEN and 2-acetylaminofluorene (2-AAF), kaempferol inhibited rat hepatocyte activity, increased apoptotic cell proportion, caspase 3 enzyme activity, ROS production, and cytochrome C release into the cytoplasm, suggesting its role in enhancing ROS production and regulating oxidative stress to induce apoptosis in HCC cells. Furthermore, Tan et al. (Tan et al., 2024) found that ellagic acid can overcome sorafenib resistance in HCC by targeting the MAPK and Akt/mTOR signaling pathways. Treatment with ellagic acid resulted in decreased cell viability in Huh7 and Hep3B cells, increased apoptosis ratio, and smaller tumor volumes and weights in nude mice with subcutaneously transplanted tumors. The study observed an increase in apoptotic proteins c-PARP, c-caspase 3, and BAX. Transcriptomics and KEGG analysis suggested suppression of the MAPK and Akt/mTOR pathways, as evidenced by decreased phosphorylation levels of ERK, JNK, MAPK, Akt, and mTOR proteins. Additionally, the combined treatment of ellagic acid and sorafenib displayed enhanced anti-tumor effects, highlighting the importance of targeting MAPK and Akt/mTOR signaling pathways for improved therapeutic results. Li et al. (Li et al., 2019) conducted research on the anti-tumor efficacy of TCM compound CP, both *in vitro* and *in vivo*. *In vitro* experiments showed that *P. urinaria* could inhibit the proliferation, migration, and colony formation of HepG2 cells. *In vivo* studies demonstrated a dose-dependent inhibition of subcutaneously transplanted tumors in mice by *P. urinaria*. The research also indicated a reduction in mRNA and protein expression of HBx, PTCH-1, SMO, GLI-1, and GLI-2, suggesting that *P. urinaria* delays the progression of HBV-related HCC by inhibiting the HBx-SHH pathway.

Zhao et al. (Zhao et al., 2008) demonstrated that corilagin dose-dependently inhibited the inflammatory response of macrophages RAW264.7 induced by LPS. This inhibition led to reduced secretion of TNF-α, IL-1β, IL-6, NO, and IL-10, along with decreased mRNA and protein expression of NF-κB and COX-2. Conversely, corilagin increased the expression of the anti-inflammatory enzyme heme oxygenase-1 (HO-1). These findings suggest that *P. urinaria* possesses anti-inflammatory properties, can prevent macrophage M2 type polarization through the NF-κB signaling pathway, promote M1 type transformation, and provide resistance against HCC. Wan et al. (Wan et al., 2019) found that Qizhu Decoction, containing seven herbal ingredients including *P. urinaria*, effectively treated DEN-induced HCC. Primary hepatocytes isolated from the HCC model and treated with Qizhu Decoction revealed corilagin as the primary metabolite in the extract. CCK8 and cell colony formation assays showed that Qizhu Decoction suppressed HCC cell proliferation. Wound healing experiments indicated reduced cell migration with Qizhu Decoction treatment. Flow cytometry analysis showed an increase in apoptotic cells. Western blot analysis demonstrated elevated expression levels of EMT marker proteins E-cadherin and caspase 3, while N-cadherin and Bcl-2 expression decreased. Additionally, both mRNA and protein levels of NF-κB signaling pathway components p-p65, TNF-α, IL-1β, and COX-2 were downregulated, indicating that *P. urinaria* exerts its anti-HCC effects by modulating the NF-κB signaling pathway.

Qizhu Anticancer Recipe (QACR) is a herbal compound containing ten botanical drugs, including *P. urinaria*. Studies indicate that QACR can hinder the proliferation of HCC cells by targeting the c-Jun N-terminal kinase signaling pathway (Han Z. et al., 2023). *In vitro* studies, QACR has been shown to inhibit the growth of MHCC97-L and SK-Hep-1 cells, induce cell apoptosis, reduce levels of PCNA, Bcl-2, and CD31 proteins, enhance expression of apoptosis-related proteins like caspase 3, caspase 8, caspase 9, and DFF40, and decrease HUVEC cell angiogenesis. Experiments on orthotopic HCC mouse models with MHCC97-L cells revealed that QACR treatment can suppress tumor growth. The protein expression patterns observed *in vivo* align with the *in vitro* results, with QACR treatment resulting in increased p-JNK expression. Additionally, using the signaling pathway inhibitor SP600125 can reverse the effects on p-JNK expression induced by QACR treatment. High-resolution mass spectrometry identified metabolites of QACR, distributed in subcutaneously transplanted tumors of nude mice post oral administration, including Deacetyl asperulosidic acid methyl ester, paeoniflorin, liquiritin, and glycyrrhizic acid. Molecular docking, network pharmacology analysis, and western blot experiments confirmed the efficacy of QACR in HCC treatment. Potential targets of QACR in HCC were identified as VEGFA, TERT, ABCB1, CA9, JUN, AR, MMP7, MMP1, and ESR2. Both *in vivo* and *in vitro* experiments demonstrated that QACR can inhibit cell proliferation, slow tumor growth in nude mice, and arrest the cell cycle at the G0/G1 phase. These results suggest that QACR exerts its anti-HCC effects through the ERK/Akt pathways (Lu et al., 2013).

#### 3.5.7 Clinical applications

In a clinical trial conducted by Tong et al. (Tong et al., 2014), the study investigated the effects of TCM Compound CP on patients with HBV-related cirrhosis. The treatment group, consisting of 52 patients, received additional treatment with CP over a 3-year observation period. Results showed that *P. urinaria* effectively inhibited liver disease progression and delayed the development of HBV-related cirrhosis to HCC. Post-treatment, reductions in HBV-DNA levels, lower AFP levels compared to the control group, decreased precancerous serum markers URG11 (β-catenin) and URG19 (vascular endothelial growth factor receptor 3, VEGFR3), and increased DRG2 (Suil) were observed. During the 5 year follow-up, only 3 new cases of HCC were reported in the treatment group, while 15 cases occurred in the control group. These findings suggest that *P. urinaria* may enhance the immune system by inducing cell cycle arrest in HCC cells, inhibiting angiogenesis, and impeding the progression of HCC.

## 4 Discussion

A thorough examination of toxicological effects is imperative to ensure the safety and efficacy of clinical medication, conducting toxicity tests on extracts and metabolites is essential to guarantee the clinical efficacy of *P. urinaria,* addressing issues related to heavy metals, pesticide residue, and mycotoxin is crucial during toxicity testing. Moreover, there has been no extensive exploration into the specificity of different metabolites in treating liver disease and their distinct impact on its progression, lack of a dearth of comprehensive investigations into the mechanism of action of the various metabolites of *P. urinaria*. To enhance the quality and therapeutic efficacy of *P. urinaria*, it is imperative to improve the extraction, purification, and preparation processes of *P. urinaria*, more stringent quality control standards should be implemented to mitigate the occurrence of adverse reactions. It is crucial to develop sensitive analytical methodologies to test the metabolites of *P. urinaria* and ensure their quality under various conditions like picking time, geographical regions, and other factors. Clinical studies are scarce on the treatment of liver disease, with a notable absence of high-quality clinical trials to substantiate its safety and efficacy. Existing clinical studies primarily consist of observational research, lacking sufficient observational indicators and robust experimental design. Prior to initiating randomized, placebo-controlled, double-blind clinical trials, it is essential to have sufficient pre-clinical testing data on *P. urinaria* and its metabolites. Furthermore, more studies are required to elucidate the relationship between the monomer metabolites and effective parts in *P. urinaria*’s disease treatment, offering insights for drug development and application. Additional research on anti-inflammatory, antibacterial, and other aspects is crucial to support its clinical use. Existing literature on anti-liver fibrosis/cirrhosis shows consistency in most basic experimental detection indicators. However, the clinical study of liver fibrosis treatment targeting HSCs is lacking, highlighting the need for an effective integration of modern treatment and ethnic medicine. Limited research exists on the use of *P. urinaria* in treating liver cirrhosis, a critical stage in the progression from liver fibrosis to HCC, underscoring the necessity for further investigation. Further research is needed to fully understand the mechanisms behind liver protection and antioxidant effects to meet clinical demands. Utilizing computational chemistry techniques to study the structural modification of active metabolites in plant extracts is essential for future investigations. It is important to recognize that the clinical benefits are not solely attributed to a single metabolite, thus combining various extracts from *P. urinaria* may enhance the overall therapeutic effect.

## 5 Conclusion and future perspectives

A comprehensive review of the pharmacological and clinical effects of *P. urinaria* on viral hepatitis, liver fibrosis/cirrhosis, and HCC has demonstrated its anti-viral, anti-tumor, and liver-protective activities through basic experiments and clinical applications. In future research, it is recommended to combine modern techniques such as proteomics and transcriptomics to investigate the impact of *P. urinaria* metabolites on gene expression and signal pathways in immune cells and liver cells. This will help elucidate the role of these changes in the progression of liver disease. Furthermore, future randomized controlled trials and multi-center studies should be conducted to assess the therapeutic potential of *P. urinaria* in liver disease. The low metabolic stability and bio-availability of metabolites can limit their effectiveness. Further clinical evaluation of drug delivery strategies like nanoparticles, liposomes, and polymeric micelles is needed to address these limitations. Standardization is crucial in the drug development process, including exploring various dosage forms like pills, tablets, granules, and capsules. Developing new technologies and optimizing existing ones can enhance metabolite yield. *P. urinaria*, as an ethnic medicine, shows therapeutic potential and may serve as a natural product with minimal adverse reactions compared to modern medicine. Accurate identification and appropriate preparation methods are essential for utilizing *P. urinaria* and other species effectively. It is recommended to establish metabolite fingerprints for batch-to-batch quality control. Detailed studies on the anti-viral, immune tolerance regulation and liver fibrosis improvement mechanisms of *P. urinaria* are needed. Researchers should also consider the synergistic effects of *P. urinaria* with anti-viral, liver protection, and anti-tumor drugs, and explore the optimal dosage and sequence of administration in combination therapies. Additionally, documenting the therapeutic outcomes of *P. urinaria* in different disease stages and populations is essential for ensuring its safety and minimizing adverse reactions. It is important to identify the key metabolites responsible for their anti-tumor effects, evaluate the impact of different solvent extracts on efficacy and mechanism, and conduct additional clinical trials to assess *P. urinaria*’s therapeutic effects. These steps are crucial for enhancing its clinical efficacy and promoting widespread application.

## Abbreviations

HCC, Hepatocellular Carcinoma; *P. urinaria*, *Phyllanthus urinaria* L.; HSCs, Hepatic Stellate Cells; HBV, Hepatitis B Virus; HCV, Hepatitis C Virus; NAFLD, Nonalcoholic fatty liver disease; HBx, Hepatitis B virus x Protein; EBV, Epstein-Barr virus; ALT, Alanine aminotransferase; AST, Aspartate aminotransferase; IGS, Intergenic spacer; HPLC, High performance liquid chromatography; SIMCA, Soft independent modeling of class analogy; TCM, Traditional Chinese Medicine; CHB, Chronic Hepatitis B; HBeAg, Hepatitis Be antigen; HBsAg, Hepatitis B surface antigen; TNF-α, Tumor Necrosis Factor Alpha; TGF-β, Transforming Growth Factor Beta; ERK, Extracellular signal-regulated kinase; STAT, Signal Transducer and Activator of Transcription; Shh, Sonic Hedgehog; TGF, Transforming growth factor; TLR4, Toll-like receptor 4; ECM, Extracellular matrix; IL-6, Interleukin 6; IL-10, Interleukin-10; IL-17, Interleukin 17; IL-1β, Interleukin 1β; Cyt-c, Cytochrome c; DEN, Diethylnitrosamine; DNA, Deoxyribonucleic Acid; CAT, Cationic amino acid transporter protein; NF-κB, Nuclear Factor Kappa-Light-Chain-Enhancer of Activated B Cells; PI3K, Phosphoinositide 3-Kinase; SOD, Superoxide Dismutase; VEGF, Vascular Endothelial Growth Factor; VEGFR-2, Vascular Endothelial Growth Factor Receptor 2; α-SMA, Alpha Smooth Muscle Actin; HepG2, Hepatoma G2; EGF, Endothelial Growth Factor; GSH, Glutathione; MAPK, Mitogen-Activated Protein Kinase; MMP, Matrix Metalloproteinases; PPAR, Peroxisome proliferator-activated receptor; HMGB1, High mobility group box 1 protein; SMO, Smoothened; PTEN, Phosphatase and tensin homology deleted on chromosome ten; TLR4, Toll-like receptor 4; SD rats, Sprague Dawley rats; TAA, Thioacetamide; CCL4, Carbon tetrachloride; PLGA, Polylactic-co-glycolic acid; ROS, Reactive oxygen species; EMT, Epithelial-to-mesenchymal transition; MET, Mesenchymal-to-epithelial transition.
