# Supplementary material for: A review of Phyllanthus urinaria L. in the treatment of liver disease: viral hepatitis, liver fibrosis/cirrhosis and hepatocellular carcinoma
Source: Front Pharmacol. 2024 Aug 9;15:1443667. doi: 10.3389/fphar.2024.1443667 (PMC11341462; doi:10.3389/fphar.2024.1443667)
Supplement: Supplementary file 1 [file DataSheet1.pdf]

Supplementary material

A review of *Phyllanthus urinaria* L. in the treatment of liver disease: viral hepatitis, liver fibrosis/cirrhosis and hepatocellular carcinoma

Linhua Liu 1,2†, Bing Wang 1†, Yibo Ma 1, Kunhui Sun 1, Ping Wang 1, Meifang Li 1, Junlin Dong 1, Meirong Qin 1, Mingshun Li 3, Chunshan Wei 3, Ying Tan 2*, Jinsong He 3*, Keying Guo 4*, Xie-an Yu 1*

1 NMPA Key Laboratory for Quality Research and Evaluation of Traditional Chinese Medicine, Shenzhen Institute for Drug Control, Shenzhen 518057, China.
2 State Key Laboratory of Chemical Oncogenomics， Institute of Biopharmaceutical and Health Engineering, Shenzhen International Graduate School, Tsinghua University, Shenzhen, China
3 Department of Biotechnology and Food Engineering, Guangdong-Technion Israel Institute of Technology, Shantou, 515063, China.
4 Department of Liver Disease, Shenzhen Traditional Chinese Medicine Hospital, The Fourth Clinical Medical College of Guangzhou University of Chinese Medicine, Shenzhen 518033, China.

* Corresponding author:

Xie-an Yu, Shenzhen Institute for Drug Control, Shenzhen 518057, China. E-mail address: yuxieanalj@126.com

Keying Guo, Guangdong-Technion Israel Institute of Technology, Shantou, 515063, China. E-mail address: keying.guo@gtiit.edu.cn

Jinsong He, Shenzhen Traditional Chinese Medicine Hospital, The Fourth Clinical Medical College of Guangzhou University of Chinese Medicine,
Shenzhen 518033, China. E-mail address: hjsprayforyou@sina.com

Ying Tan, State Key Laboratory of Chemical Oncogenomics, Institute of Biomedical and Health Engineering, Shenzhen International Graduate School, Tsinghua University, Shenzhen, 518055, China. E-mail address: tan.ying@sz.tsinghua.edu.cn

Supplementary Tables

Table S1 Isolation and information of metabolites from *Phyllanthus urinaria* L.

	Metabolites	CAS NO.	Molecular formula	Molecular weigh	References
Flavonoids					
	Rhamnocitrin	569-92-6	$C_{16}H_{12}O_6$	300.26	Qi et al., 2014, Fang et al., 2008
	Quercetin 3-O-β-D-glucoside	21637-25-2	$C_{21}H_{20}O_{12}$	464.376	Qi et al., 2014, Xu et al., 2007
	Quercetin 3-rutinoside	949926-49-2	$C_{27}H_{30}O_{16}$	610.518	Fang et al., 2008
	Rutin	153-18-4	$C_{27}H_{30}O_{16}$	610.518	Qi et al., 2014, Fang et al., 2008, Xu et al., 2007
	Isoquercitrin	482-35-9	$C_{21}H_{20}O_{12}$	464.376	Mao et al., 2016
	Quercetin 3-O-α-L-rhamnopyranoside	1269988-72-8	$C_{22}H_{22}O_{11}$	462.403	Mao et al., 2016, Wu et al., 2013
	Quercetin 3-(4'-acetylrhamnoside) 7-rhamnoside	17306-45-5	$C_{27}H_{30}O_{16}$	610.52	Han et al., 2023a
	Epigallocatechin	970-74-1	$C_{15}H_{14}O_7$	306.267	Han et al., 2023a
	Astragalin	480-10-4	$C_{21}H_{20}O_{11}$	448.377	Calixto et al., 1998
	Quercetin	117-39-5	$C_{15}H_{10}O_7$	302.236	Calixto et al., 1998, Wang and Lee, 2005, Wu et al., 2013
	Quercitrin	522-12-3	$C_{21}H_{20}O_{11}$	448.377	Calixto et al., 1998, Fang et al., 2008
	Kaempferol	520-18-3	$C_{15}H_{10}O_6$	286.236	Calixto et al., 1998
	Urinariaflavone	/	$C_{18}H_{16}O_9S$	407.04368	Thanh et al., 2014
	Quercetin 3-*O*-α-L-(2,4-di-*O*-acetyl) rhamnopyranoside-7-*O*-α-L-rhamno pyranoside	/	$C_{31}H_{34}O_{17}$	679.1874	Wu et al., 2013
	Quercetin 3-*O*-α-L-(3,4-di-*O*-acetyl) rhamnopyranoside-7-*O*-α-L-rhamno pyranoside	/	$C_{31}H_{34}O_{17}$	679.1874	Wu et al., 2013
Lignans					
	Phyltetralin	123048-17-9	$C_{24}H_{32}O_6$	416.51	Qi et al., 2014, Fang et al., 2008, Wang and Lee, 2005, Chang et al., 2003
	Heliobuphthalmin lactone	580-73-4	$C_{20}H_{18}O_6$	354.35332	Qi et al., 2014, Chang et al., 2003
	Hypophyllanthin	33676-00-5	$C_{24}H_{30}O_7$	430.491	Sarin et al., 2014, Wang and Lee, 2005, Fan et al., 2015, Chang et al., 2003
	Nirtetralin	50656-78-5	$C_{24}H_{30}O_7$	430.491	Sarin et al., 2014, Wang and Lee, 2005, Fan et al., 2015, Chang et al., 2003
	Dihydrocubebin	24563-03-9	$C_{20}H_{22}O_6$	358.385	Hu et al., 2014
	(7S,7'S,8R,8'R)-icariol A2	161657-71-2	$C_{22}H_{28}O_9$	436.452	Hu et al., 2014
	Evofolin B	1961305-60-1	$C_{17}H_{18}O_6$	318.321	Hu et al., 2014
	Episyringaresinol	51152-20-6	$C_{22}H_{26}O_8$	418.44	Hu et al., 2014
	Boehmenan	57296-22-7	$C_{40}H_{40}O_{12}$	712.74	Han et al., 2023a
	Isolintetralin	145459-30-9	$C_{23}H_{28}O_6$	400.465	Mao et al., 2016, Chang et al., 2003

	Lintetralin	73231-44-4	$C_{23}H_{28}O_6$	400.465	Mao et al., 2016, Wang and Lee, 2005, Chang et al., 2003
	Niranthin	50656-77-4	$C_{24}H_{32}O_7$	432.507	Mao et al., 2016, Wang and Lee, 2005, Fan et al., 2015,
	Phyllanthin	10351-88-9	$C_{24}H_{34}O_6$	418.523	Mao et al., 2016
	Virgatusin	176050-44-5	$C_{23}H_{28}O_7$	416.46	Wang and Lee, 2005, Chang et al., 2003
Tannins					
	Corilagin	23094-69-1	$C_{27}H_{22}O_{18}$	634.453	Mao et al., 2016, Xu et al., 2007, Zhong et al., 2013, Jikai et al., 2002, He et al., 2022
	Geraniin	60976-49-0	$C_{41}H_{28}O_{27}$	952.645	Mao et al., 2016, Zhong et al., 2013
	Hippomanin A	52934-78-8	$C_{27}H_{22}O_{18}$	634.453	Mao et al., 2016
	Isostrictinin	84316-77-8	$C_{27}H_{22}O_{18}$	634.453	Mao et al., 2016, Han et al., 2023a
	Phyllanthusiin A	142705-58-6	$C_{41}H_{28}O_{27}$	952.645	Han et al., 2023a
	Phyllanthusiin D	133145-19-4	$C_{44}H_{32}O_{27}$	992.709	Han et al., 2023a
	1,3,4,6-tetra-O-galloyl-β-D-glucose	26922-99-6	$C_{34}H_{28}O_{22}$	788.58	Zhong et al., 2013, Yang et al., 2007ab, Yang et al., 2007b
	Gemin D	84744-46-7	$C_{27}H_{22}O_{18}$	634.453	Zhong et al., 2013
Phenolic acids					
	Furosin	81552-37-6	$C_{27}H_{22}O_{19}$	650.452	Xu et al., 2007
	Mallotinin	125445-51-4	$C_{41}H_{30}O_{28}$	970.66	Xu et al., 2007
	Repandusinic acid A	125516-10-1	$C_{41}H_{30}O_{28}$	970.66	Xu et al., 2007
	Ferulic acid	1135-24-6	$C_{10}H_{10}O_4$	194.184	Hu et al., 2014, Deng et al., 2012
	Repandinin B	/	/	/	Xu et al., 2007
	Dehydrochebulic acid trimethyl ester	154702-77-9	$C_{17}H_{16}O_{11}$	396.302	Hu et al., 2014
	3,5-Dihydroxy-4-methoxybenzoic acid	4319-02-2	$C_8H_8O_5$	184.146	Mao et al., 2016, Hu et al., 2014
	Methyl gallate	99-24-1	$C_8H_8O_5$	184.146	Qi et al., 2014, Fang et al., 2008
	Protocatechuic acid	99-50-3	$C_7H_6O_4$	154.12	Qi et al., 2014, Xu et al., 2007
	2,3,4,5,6-Pentahydroxybenzoic acid	145279-23-8	$C_7H_6O_7$	202.118	Qi et al., 2014
	Syringin	118-34-3	$C_{17}H_{24}O_9$	372.367	Qi et al., 2014, Xu et al., 2007
	Brevifolin carboxylic acid	18490-95-4	$C_{13}H_8O_8$	292.198	Qi et al., 2014, Xu et al., 2007
	3,3’,4’-Tri-O-methylellagic acid	5145-53-9	$C_{17}H_{12}O_8$	344.27	Qi et al., 2014
	Ellagic acid	476-66-4	$C_{14}H_6O_8$	302.193	Qi et al., 2014, Jikai et al., 2002, Guo et al., 2021
	Phyllanthurinolactone	168180-12-9	$C_{14}H_{18}O_8$	314.288	Qi et al., 2014, Mao et al., 2016, Urakawa et al., 2004
	Brevifolin	477-94-1	$C_{12}H_8O_6$	248.188	Mao et al., 2016
	Gentisic acid 4-O-β-D-glucopyranoside	1820-89-9	$C_{13}H_{16}O_9$	316.261	Xu et al., 2007
	p-Hydroxybenzaldehyde	123-08-0	$C_7H_6O_2$	122.121	Mao et al., 2016, Hu et al., 2014
	Pyrogallol	87-66-1	$C_6H_6O_3$	126.11	Mao et al., 2016
	Hexacosanoic acid	506-46-7	$C_{26}H_{52}O_2$	396.69	Calixto et al., 1998
	Gallic acid	149-91-7	$C_7H_6O_5$	170.12	Calixto et al., 1998, Xu et al., 2007

	Gallic acid ethyl ester	831-61-8	$C_9H_{10}O_5$	198.17	Santos et al., 1999, Paulino et al., 1999
	Caffeic acid	331-39-5	$C_9H_8O_4$	180.16	Paulino et al., 1999
	Ascorbic Acid	299-36-5	$C_6H_7O_6$	175.116	Xu et al., 2007
	Arbutin	497-76-7	$C_{12}H_{16}O_7$	272.251	Xu et al., 2007
	Cucurbic acid	131488-83-0	$C_{12}H_{20}O_3$	212.285	Hu et al., 2014
	α-Cucurbic acid methyl ester	62653-84-3	$C_{13}H_{22}O_3$	226.312	Hu et al., 2014
Terpenoids					
	Lupeol acetate	1617-68-1	$C_{32}H_{52}O_2$	468.7541	Qi et al., 2014
	Lupeol	545-47-1	$C_{30}H_{50}O$	426.717	Mao et al., 2016
	β-Amyrin	559-70-6	$C_{30}H_{50}O$	426.72	Mao et al., 2016
	Glochidiol	6610-56-6	$C_{30}H_{50}O_2$	442.72	Mao et al., 2016, Hu et al., 2014
	Lup-20(29)-en-3β-ol	33869-84-0	$C_{30}H_{50}O$	426.717	Mao et al., 2016
	Cloven-2β,9α-dio	2649-64-1	$C_{15}H_{26}O_2$	238.36574	Mao et al., 2016, Hu et al., 2014
	Cleistanthol	24465-21-2	$C_{20}H_{28}O_3$	316.43	Mao et al., 2016, Hu et al., 2014
	Spruceanol	72963-56-5	$C_{20}H_{28}O_2$	300.435	Mao et al., 2016, Hu et al., 2014
	Syringaresinol	6216-81-5	$C_{22}H_{26}O_8$	418.437	Mao et al., 2016
	Dendranthemoside B	/	$C_{19}H_{32}O_8$	388.1784	Mao et al., 2016
	Betulin	473-98-3	$C_{30}H_{50}O_2$	442.717	Ueda et al., 1998
	β-betulinic acid	472-15-1	$C_{30}H_{48}O_3$	456.7	Ueda et al., 1998
	Oleanolic acid	508-02-1	$C_{30}H_{48}O_3$	456.7	Ueda et al., 1998
	28-norlup-20(29)-ene-3β,17β-diol	52591-08-9	$C_{30}H_{50}O_2$	442.717	Ueda et al., 1998
m					
	Montanoic acid ethyl ester	55682-92-3	$C_{29}H_{58}O_2$	438.77	Calixto et al., 1998
	Methyl brevifolin carboxylate	154702-76-8	$C_{14}H_{10}O_8$	306.224	Calixto et al., 1998, Fang et al., 2008
Sterols					
	(3β,22E)-Stigmasta-5,22-diene-3,25-diol	64998-19-2	$C_{29}H_{48}O_2$	428.69	Mao et al., 2016
	Daucosterol	474-58-8	$C_{35}H_{60}O_6$	576.847	Mao et al., 2016
	Stigmasterol	83-48-7	$C_{29}H_{48}O$	412.691	Mao et al., 2016
	Stigmasterol 3-O-β-D-glucoside	5041-82-7	$C_{22}H_{22}O_{12}$	478.403	Mao et al., 2016
	β-Sitosterol	83-46-5	$C_{29}H_{50}O$	414.707	Mao et al., 2016, Hu et al., 2014
	β-Sitosterol-3-O-β-D-glucopyranoside	1131372-16-1	$C_{35}H_{60}O_6$	576.847	Mao et al., 2016, Hu et al., 2014
Alkaloids					
	Phyllurine	/	/	/	Ueda et al., 1998
	Triacontanol	28351-05-5	$C_{30}H_{62}O$	438.813	Calixto et al., 1998
Others					
	Menisdaurin	67765-58-6	$C_{14}H_{19}NO_7$	313.303	Qi et al., 2014
	5-hydroxymethyl-2-furaldehyde	67-47-0	$C_6H_6O_3$	126.11	Hu et al., 2014
	Epigallocatechin-(4β->8)-catechin	77983-30-3	$C_{30}H_{26}O_{13}$	594.52	Han et al., 2023a
	Phthalic acid bis-ester	117-83-9	$C_{20}H_{30}O_6$	366.45	Satyan et al., 1995

Table S2 The metabolites from *Phyllanthus urinaria* L. for anti-Viral Hepatitis, and the mechanism

Extract used	Metabolites	Dosage	IC_{50}	Methods	Applied	Mechanism	Effects	References
Ethanol	Corilagin		2.28 μM	Molecular docking	Corilagin bindng site of HCV	Interfere of HCV NS3 serine protease	Anti-HCV	Yue et al., 2005
Acetone	Loliolide		1.5±0.2 μg/mL	*In vitro*	Huh 7.5 cells were infected with HCVcc	Inactivating virus particles	Anti-HCV	Chung et al., 2016a
Acetone	(4R,6S)-2-Dihydromenisdaurilide		14.25±2.6 μM	*In vitro*	Huh 7.5 cells were infected with HCVcc	Preventing HCV entry into cells during the initial infection stage	Anti-HCV	Chung et al., 2016b
Aqueous	The whole plant	5 mg/kg/day, duration 30 days		*In vivo*	DHBV-infected ducks	Interfere in plasma HBV DNA level	Anti-HBV	Chen and Zhu, 2013
Aqueous	Ellagic acid		0.07 μg/mL	*In vitro*	HepG2 2.2.15 cell	Reduced HBeAg secretion	Anti-HBV	Shin et al., 2005
Aqueous	Ellagic acid	5 mg/kg/day, duration 2 weeks		*In vivo*	HBeAg producing transgenic mice	Boost immune responses, decrease HBeAg levels, and enhance cytokine release	Anti-HBV	Kang et al., 2006
Aqueous	Ellagic acid		0.8 and 0.2 g/L	*In vitro*	Plasmid pHBV1.1 into HepG2 cells	Reduced HBsAg secretion	Anti-HBV	Wu et al., 2015
Ethanol	Emodin-8-O-β-D-glucopyranoside, catechin, 3-O-methylgallic acid, ethyl gallate, and protocatechuic acid		5.12 and 8.13 μM	*In vitro*	HepG2.2.15 cells	Decrease in HBsAg and HBcAg levels, decreased IL-6 mRNA expression, alter the ERK1/2 and JNK signaling pathways	Anti-HBV	Liang et al., 2019
Aqueous	Gallic acid, kaempferol,		250 μg/mL	*In vitro*	HepG2 2.2.15 hepatoblastoma cell	Reduce HBsAg, HBeAg, and HBV DNA secretion, increased the expression of NFE2L2	Anti-HBV	Fu et al., 2023

	quercetin, corilagin, and rutin				and HMOX1 proteins		
Aqueous	The whole plant	30 g, duration 96 weeks	Clinical application	HBeAg positive with normal ALT patients	High rates of HBV DNA reduction	Anti-HBV	Xing et al., 2020
Ethanol	The whole plant	1, 2, 3 g, duration 6 months	Clinical application	Positive HBeAg and elevated ALT patients	HBV DNA levels descend	Anti-HBV	Chan et al., 2003

Table S3 The metabolites from *Phyllanthus urinaria* L. for anti-liver fibrosis/cirrhosis, and the mechanism.

Extract used	Metabolites	Dosage	IC_{50}	Methods	Applied	Mechanism	Effects	References
Methanol	Quercetin	5 and 15 mg/kg, duration 8 weeks		*In vivo*	Induced by CCl4 in SD rats	Reduced levels of serum markers, improved liver appearance, decreased collagen deposition, down-regulation NF-κB/p38, and MAPK/Bax expression, increased Bcl-2 levels	Inhibit HSCs activation	Wang et al., 2017
	Quercetin	0.5 mg/kg, duration 6 weeks		*In vivo*	Induced by concanavalin A in mice	Reduced expression of α-SMA, NF-κB, and TGF-β	Reduced HSCs activation	Wan et al., 2014
	Quercetin	50 mg/kg/day, duration 8 weeks	50 μM	*In vivo/ In vitro*	Mice was injected with CCl4, rat HSCs	Lower portal inflammation and fibrosis scores, decreased serum enzymes ALT, AST, and the expression of TGF-β1, α-SMA, HMGB1, TLR2/TLR4, and NF-κB p65 proteins	Reduced HSCs activation	Li et al., 2016
	Quercetin	50 mg/kg/day, duration 4 weeks		*In vivo*	SD rats injections of TAA	Increase in serum SOD levels and a decrease in the expression of Shh, Ihh, Ptch-1, Smo, Gli3, TNF-α and NF-κB	Reduced HSCs activation	Aslam et al., 2022
	Quercetin	100 mg/kg/day, duration 8 weeks		*In vivo*	CCl4 induced liver fibrosis in rats	Reducing the expression of TNF-α, IL-6, TGF-β1, COL1α1, CTGF, TIMP-1, and α-SMA, increased the expression of TGF-β1, MMP2, and MMP9	Induced HSCs apoptosis	Hernandez-Ortega et al., 2012
	Quercetin	50 mg/kg/day, duration 8 weeks	50 μM	*In vitro/ In vivo*	Induced by CCl4 in mice and Raw 264.7 cells	Decreases the expression of α-SMA, the levels of desmin and vimentin, decreases mRNA expression of TNF-α, IL-1β, IL-6, MCP-1, and Notch1	Inhibited the activation of HSCs	Li et al., 2018
	Kaempferol	10 uM/L,1 mL/day, duration 4 weeks		*In vivo*	Induced by CCl4 in mouse	Reduced expression of α-SMA and COLIα1, down-regulation of Notch/Jag1 and up-regulation of miR-26b-5p expression	Decreases the activation of HSCs	Zhou et al., 2022
	Kaempferol	10 uM/L,1 mL/day,	6.96 μM	*In vitro/ In vivo*	Induced by CCl4 in female	Reduction in serum levels of HA, LN, AST, ALT, and Smad2/3, down-regulation of the RNA and	Suppress HSCs collagen	Xu et al., 2019

		duration 4 weeks			C57BL/6 mice, HSCs from mouse liver	protein expression of COL1α1, α-SMA	synthesis	
Methanol	Gallic acid	50 mg/kg/day, duration 8 weeks	45 and 19 mg/mL at 24 and 48 h respectively	*In vitro/ In vivo*	TAA induced hepatotoxicity in rats, rat hepatic stellate cell line (HSC-T6)	Decrease α-SMA expression, and the levels of PCNA, PDGF-BB, TIMP-1, HP, and reduce collagen deposition	Reduction of HSCs proliferation/activation	El-Lakkany et al., 2019
	Ellagic acid, quercetin	100 mg/kg/day, duration 45 days		*In vivo*	TAA induced hepatotoxicity in rats	Decreased ALT, AST, ALP levels and the expression of MMP2, MMP9, increased TAC and GPX levels	Attenuation of liver function tests and fibrotic-related genes	Afifi et al., 2018
Methanol	Gallic acid	0.5, 1.0 g/kg, duration 24 hours		*In vivo*	Induced by CCl4 in rats	Decreased levels of GOT and GPT, increased levels of SOD, GSH, and GPX	Protecting against hepatotoxicity	Lee et al., 2006
	Phyllanthin	1,2,3,4 μg/mL, duration 24 hours		*In vivo*	Induced by CCl4 in mice	Reducing the levels of TNF-α, NF-κB, and TGF-β1	Decrease in ECM production and deposition	Chirdchupunseree and Pramyothin, 2010
	Phyllanthin	5 mg/kg/day, duration 30 days		*In vivo*	Induced by CCl4 in mouse	Mitigate the levels of ALT and AST, ameliorate the membrane damage, resolve the fibrotic-associated changes	Reduce collagen deposition	Krithika et al., 2019
	Phyllanthin	10 mg/kg/day, duration 30 days		*In vivo*	Induced by CCl4 in mouse	Reduce the expression of TGF-β1, ALK5, p-Smad2, p-Smad3, ALT, AST level decreased	Remodeling ECM synthesis and degradation	Krithika et al., 2015
Aqueous	The whole plant	1.16, 2.32, 4.64 g/kg/day, duration 10 weeks		*In vivo*	Induced by CCl4 in SD rats	The levels of AST, ALT, PCI, COL IV, LN, HA, and α-SMA decreased, modulation of the TNF-α/MAPK and NF-κB signaling pathways	Inhibit the activation of HSC and ECM deposition	Cai et al., 2022
	Corilagin	100		*In vivo*	Mice	Modulation of the IL-13 signaling	Reduced the	Huang et al.,

		mg/kg/day, duration 6 weeks		infected by schistosoma	pathway and suppression of GATA3 to regulate the Th1/Th2 balance	synthesis of collagen	2013
	Corilagin	1, 5, 10 mg/kg/day, duration 24 hours	*In vivo*	Rat hemorrhagic shock and resuscitation model	Reduce AST, ALT and MPO levels, decrease the CINC-1, CINC-3, ICAM-1, IL-6, and TNF-α concentrations	Liver protective effects	Liu et al., 2017
Aqueous	The whole plant	4.5 g/kg/day, duration 48 weeks	Clinical application	240 patients with severe liver fibrosis/cirrhosis and CHB	Increase the reversal rate of liver fibers and the normalization rate of ALT, reduce the inflammation of liver tissue	Improved liver pathology, reduced fibrous tissue proliferation	Xing et al., 2023

Table S4 The metabolites from *Phyllanthus urinaria* L. for anti-HCC, and the mechanism.

Extract used	Metabolites	Dosage	IC_{50}	Methods	Applied	Mechanism	Effects	References
	Quercetin	50 mg/kg/day, duration 28 days	3.213 µM	*In vivo/ In vitro*	Huh7 cell line and sorafenib-resistant cell line, cells was injected into BALB/c nude mice	Decreased expression of p-EGFR/EGFR, p-Akt/Akt, p-ERK/ERK, and Bcl-2 proteins, increasing Cleaved PARP1 and Bax expression, reduced tumor weight and volume	Impeded proliferation in cells, reversing acquired resistance to sorafenib	Zhang et al., 2024
	Corilagin	30 mg/kg/day, duration 5 weeks	23.4 µM	*In vivo/ In vitro*	SMMC7721 and Bel7402 cells, MHCC97-H cell xenografts in Balb/c nude mice	Down-regulates the expression of proteins cyclin B1 and ccdc2, and enhances the inhibition of oncogenes p21, Cip1, and p-p53, reduction in tumor mass and volume	Inhibit the proliferation of HCC cells	Ming et al., 2013
	Corilagin	15 mg/kg/day, duration 7 days		*In vivo*	Athymic nude mice were injected with Hep3B cells	AST and ALT have remained within normal ranges, suppressing NF-κB pathway	Suppress the growth of liver tumors	Hau et al., 2010
	Quercetin	50 mg/kg/day, duration 21 days;5,10,20	40 µM	*In vivo/ In vitro*	Huh7 cells and patient tumor xenografted into male NSG mice, Hep3B, Huh7 cell	Tumor sizes were smaller, tumor weight was lighter	Suppresses HCC cell invasion and metastasis	Huang et al., 2024
Aqueous	The whole plants	30 g	0.5 mg/mL	Network pharmacology and *in vitro*	HepG2 and Hep3B cell	Key target proteins like TP53, Akt1, STAT3, and MAPK, suppress the PI3K/Akt pathway	Inhibit invasion and migration of HCC cells	Wu et al., 2022
Aqueous	The whole plants	30 g	1.5 mg/mL	*In vitro*	Hep3B and HepG2 cell	Reduced levels of p-PI3K, Akt, and p-Akt proteins in cells	Suppress invasion and metastasis of HCC	Wei et al., 2024
Aqueous	The whole plants	30 g	159 µg/mL	*In vivo/ In vitro*	Zebra fish lung graft model and HepG2 cell	Attenuated the wound-healing ability of cells and reduced cell migration, inhibiting tumor spread in zebrafish	Inhibiting HCC metastasis	Huang et al., 2021
Aqueous	The whole plants	30 g	148.5 µg/mL	*In vitro*	HepG2 cell	Affecting the level of exosomal microRNAs, increased levels of proteins beclin-1 and LC3-II, and a decrease in p62 expression	Inhibiting invasion and metastasis in HCC	Liao et al., 2024
Ethyl acetate	7'-hydroxy-3',4,5,9		4.46 µM	*In vitro*	HepG2 cell	Up-regulate c-myc, down-regulate Bcl-2, activate caspase and inhibit	Inducing HepG2 cell	Giridharan et al., 2002

	,9'-penta methoxy-3,4-meth ylene dioxy					telomerase activity	apoptosis	
Hydrome thanolic	Gallic acid		445±65 µg/mL	*In vitro*	HepG2 cell	Suppress mitochondrial oxidative phosphorylation, decrease intracellular ATP levels, modulate Ca^{2+} levels, trigger mitochondrial dysfunction	Induce apoptosis in HCC cells	Chudapongse et al., 2010
	Corilagin		37.5 µM	*In vitro*	MHCC97-H, Bel-7402, SMMC-7721	Decrease in the mitochondrial membrane potential ratio, elevation proteins Cyto c, caspase 8, and P53, reduction in p-Akt and Bcl-2 protein expression, and cleavage of caspase 3, caspase 9, and PPAR	Induce apoptosis in HCC cells	Deng et al., 2018
	Kaempfe rol		100 µM	*In vitro*	HepG2 cell	Increased LDH activity, up-regulation of GRP94 and GRP78 levels, and induction of CHOP and caspase 3	Triggers HepG2 cell apoptosis	Guo et al., 2016
Aqueous	The whole plants		1.42 mg/mL	*In vitro*	HepG2 cell	Inhibited the viability of various cancer cells, increased DNA fragmentation	Induces apoptosis in HepG2 cells	Huang et al., 2004b
	Phyllanth in	30 mg/kg/day, duration 14 weeks	25 µM	*In vivo/ In vitro*	Wistar albino rats were induced by DEN; HepG2 cell	CEA, AFP, 8-OHdG, LDH, GGT, ALT, AST, and ALP showed elevated levels, the mRNA expression of TP53, caspase 3, caspase 9, and Bax increased	Induces apoptosis through the PI3K/Akt/mT OR signaling pathway	You et al., 2021
	Luteolin, Kaempfe rol		12µM/ 30 µM	*In vivo/ In vitro*	SD rats induced by DEN and 2-AAF; rat Hepatocytes	Increased apoptotic cell proportion, caspase 3 enzyme activity, ROS production, and cytochrome C release into the cytoplasm	Modulate ROS signaling pathways	Seydi et al., 2018
	Ellagic acid		5 µM	*In vivo/ In vitro*	Huh7 cells were implanted into BABL/c nude mice; Huh7, Hep3B cell	Decreased cell viability in cells, increased apoptosis ratio, smaller tumor volumes and weights, increase in proteins c-PARP, c-caspase 3, and Bax	Overcome sorafenib resistance in HCC by targeting the MAPK and Akt/mTOR signaling pathways	Tan et al., 2024

	The whole plants	30 g	144.2 μg/mL	*In vivo/ In vitro*	HepG2-HBx cells implanted into BABL/c nude mice; HepG2 cell	Inhibit the proliferation, migration, and colony formation of HepG2 cells, reduction in mRNA and protein expression of HBx, PTCH-1, SMO, GLI-1, and GLI-2	Delays the progression of HBV-related HCC by inhibiting the HBx-SHH pathway	Li et al., 2019
	Corilagin		5 μg/mL	*In vitro*	RAW 264.7 cell	Prevent macrophage M2 type polarization, promote M1 type transformation	Through the NF-κB pathway against HCC	Zhao et al., 2008
Ethanol	The whole plants	20 g/kg,duration 28 weeks;	5 μg/mL	*In vivo/ In vitro*	Male C57BL/6 mice induced by DEN; HepG2 and PLC/PRF/5 cells	Elevated levels of proteins E-cadherin and caspase 3, N-cadherin and Bcl-2 expression decreased, p-p65, TNF-α, IL-1β, and COX-2 were down-regulated	Anti-HCC by modulating the NF-κB signaling pathway	Wan et al., 2019
Aqueous	The whole plants	30 g	400 μg/mL	*In vivo/ In vitro*	MHCC97-L cells implanted into BALB/c male nude mice; MHCC97-L, SK-Hep-1 cell	Reduce levels of PCNA, Bcl-2, and CD31 proteins, enhance expression of proteins of caspase 3, caspase 8, caspase 9, and DFF40, and decrease HUVEC cell angiogenesis	Targeting the c-Jun N-terminal kinase signaling pathway	Han et al., 2023b
Aqueous	The whole plants		100 μg/mL	*In vivo/ In vitro*	BALB/c nude mice; Huh-7 and MHCC-97H cell	Inhibit cell proliferation, slow tumor growth, and arrest the cell cycle at the G0/G1 phase	Through the ERK and Akt pathways	Lu et al., 2013
Aqueous	The whole plants	30 g, 20 days per month, a follow-up period of 2 years		Clinical application	HBV-associated cirrhosis patients	HBV DNA levels declined, URG11, URG19 decreased and DRG2 increased	Delayed the development of HBV-related cirrhosis to HCC	Tong et al., 2014

Supplementary Figures

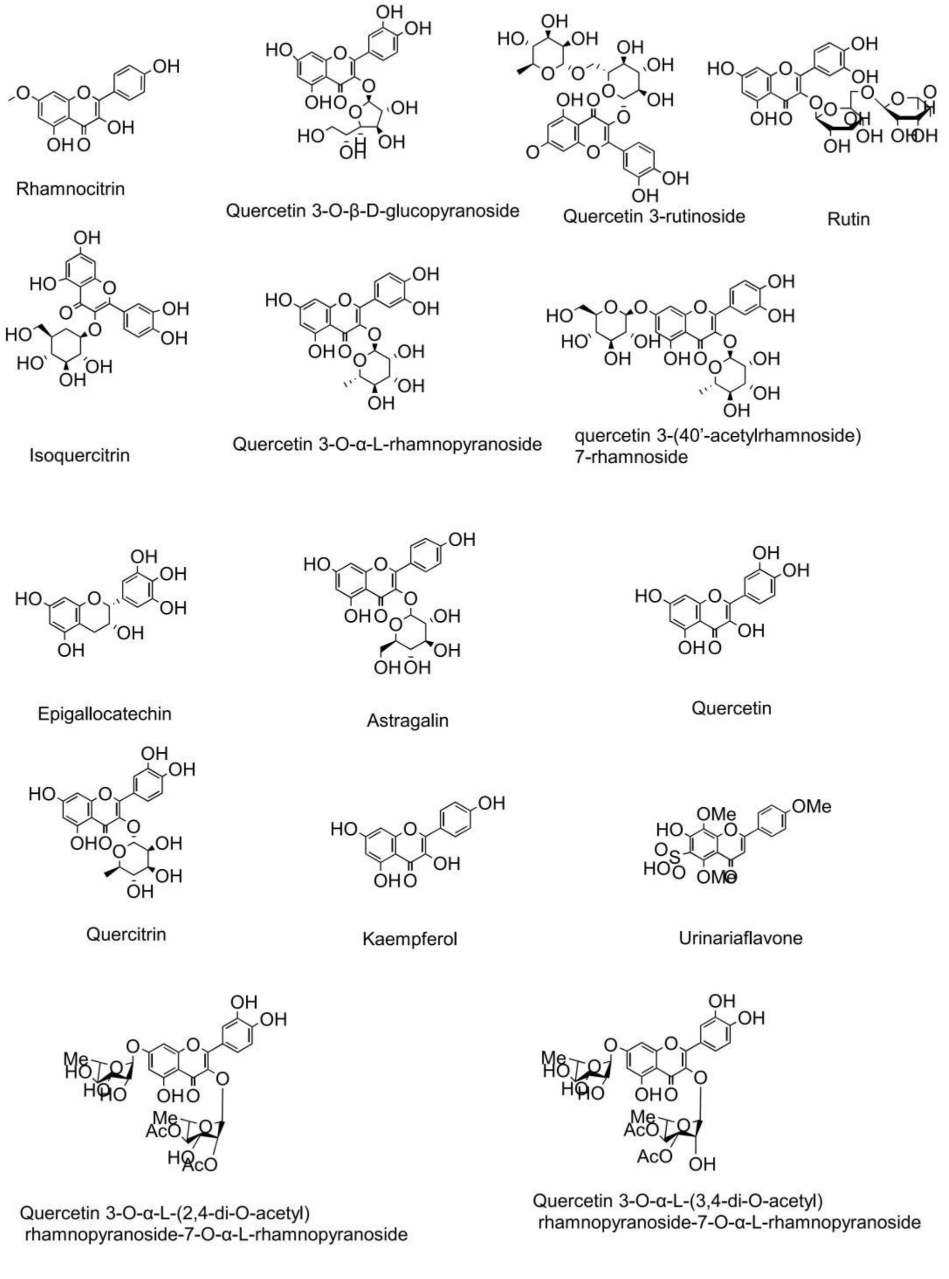

Figure S1 Chemical structure of flavonoids extracted from *Phyllanthus urinaria* L.

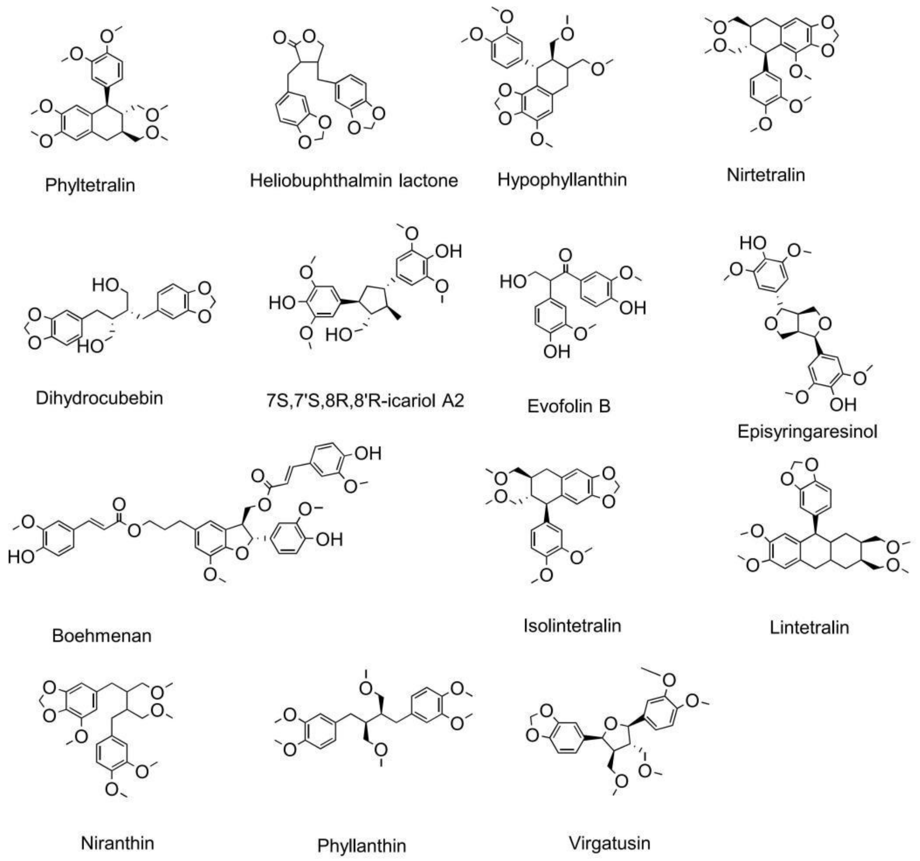

Figure S2 Chemical structure of lignans extracted from *Phyllanthus urinaria* L.

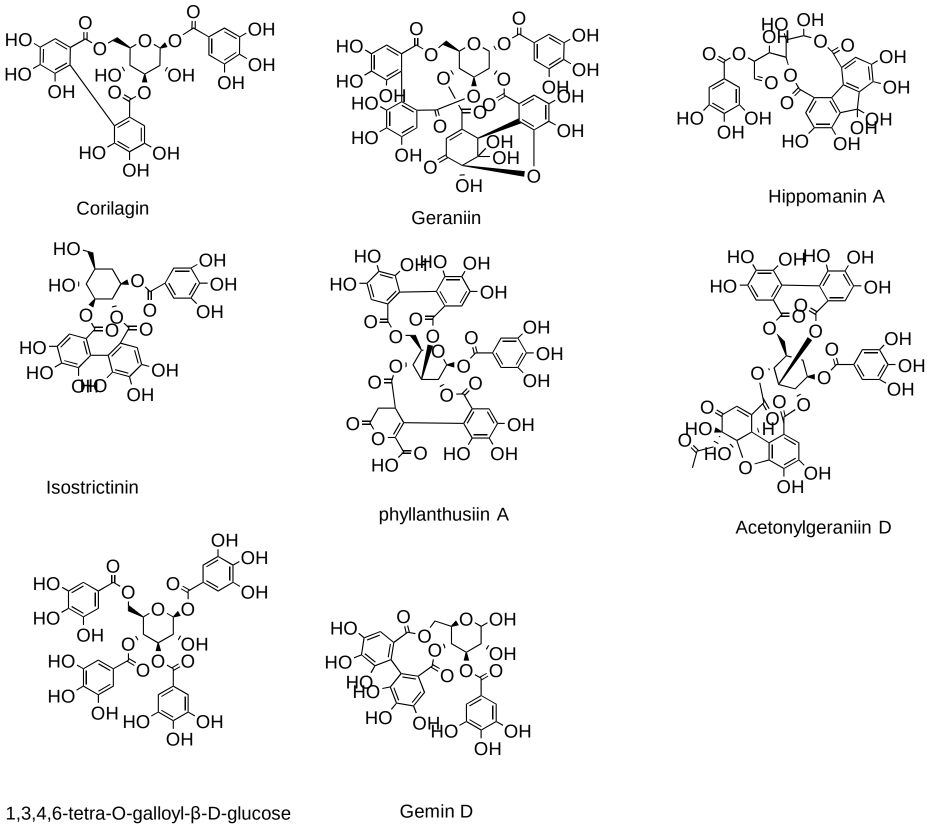

Figure S3 Chemical structure of tannins extracted from *Phyllanthus urinaria* L.

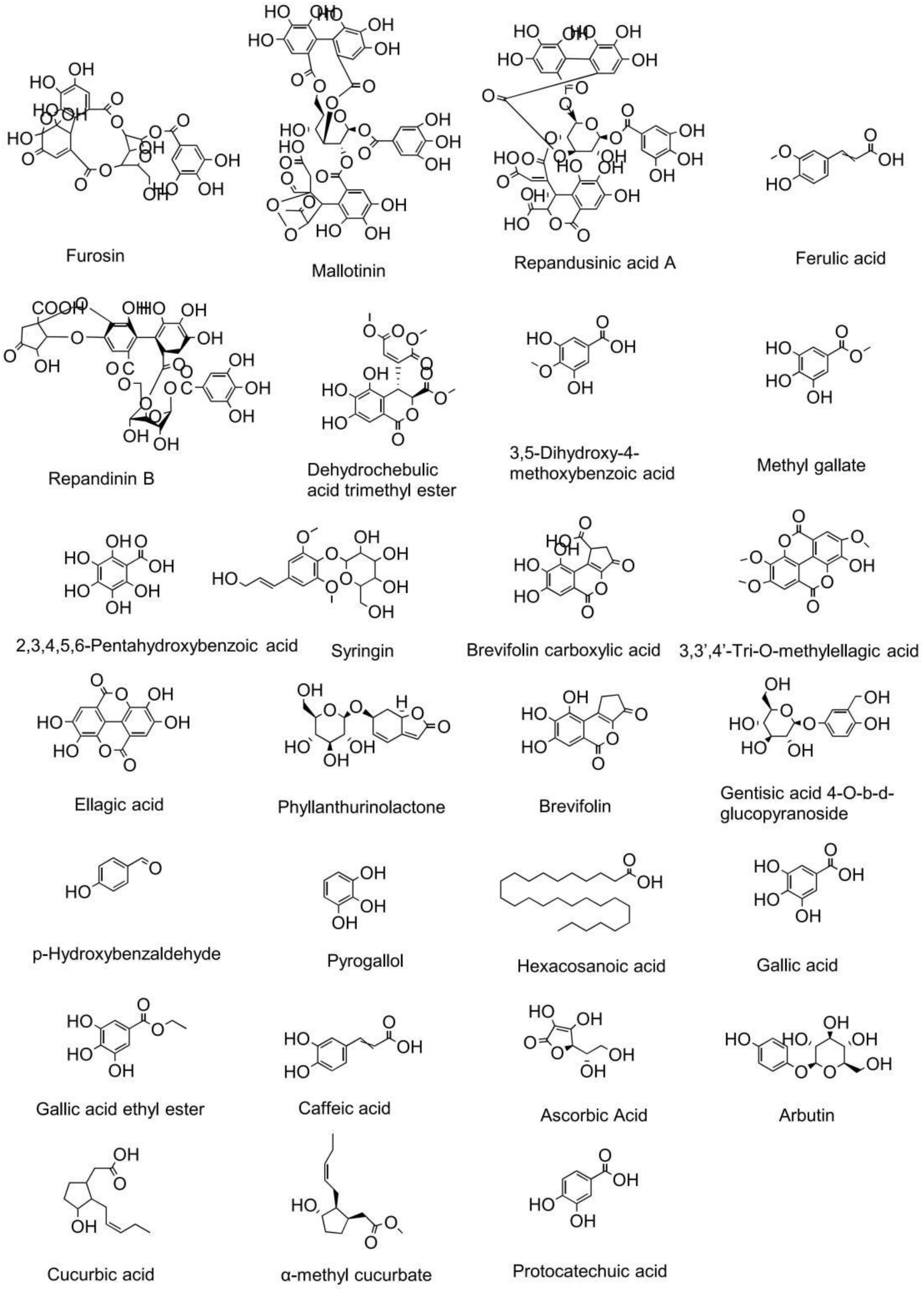

Figure S4 Chemical structure of phenolic acids extracted from *Phyllanthus urinaria* L.

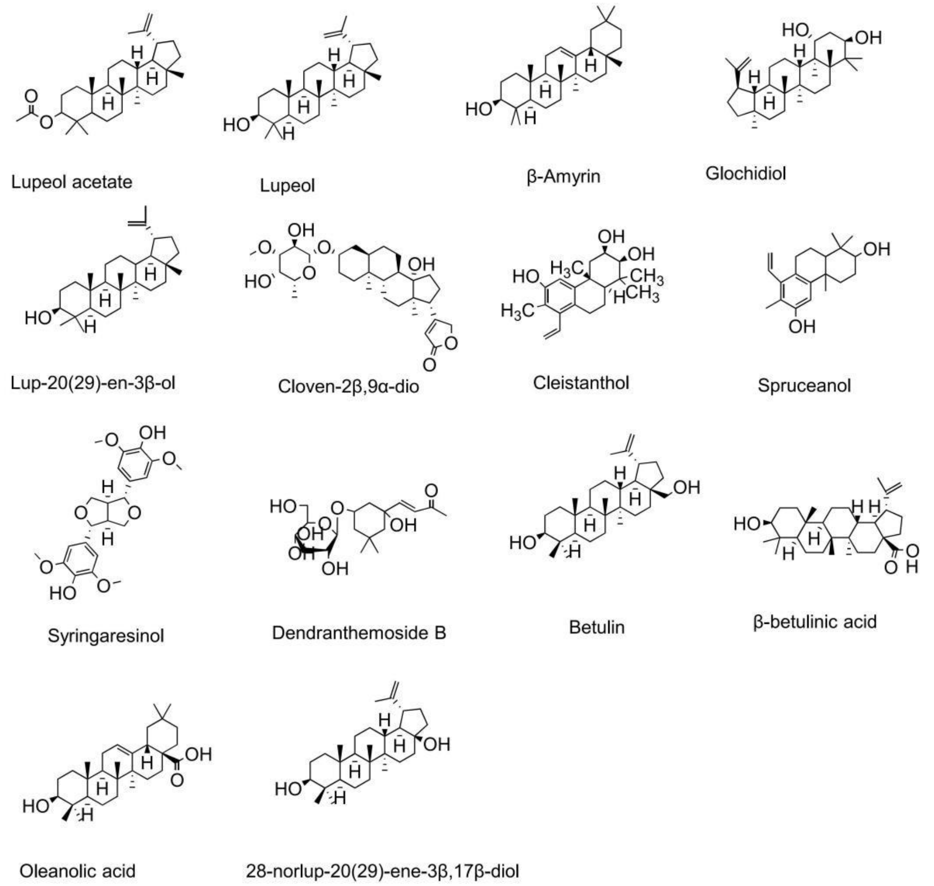

Figure S5 Chemical structure of terpenoids extracted from *Phyllanthus urinaria* L.

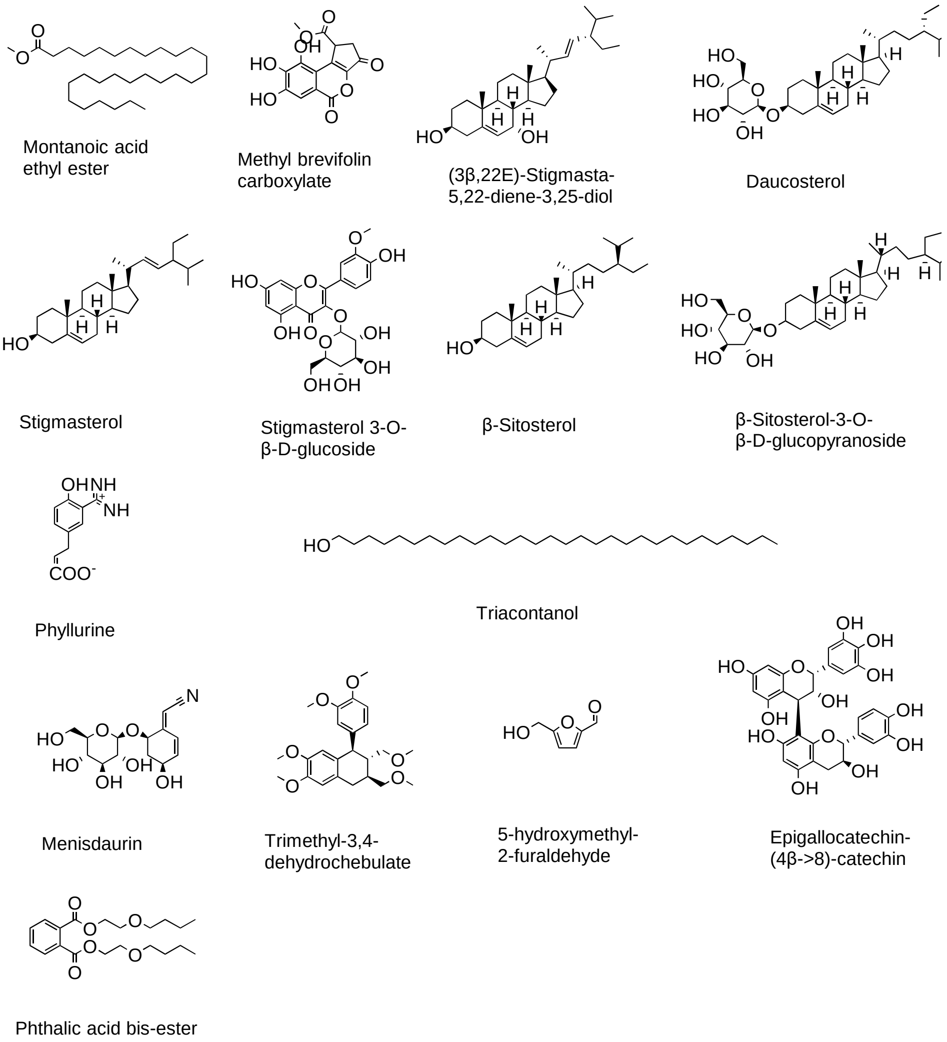

Figure S6 Chemical structure of others metabolites extracted from *Phyllanthus urinaria* L.
